# Protection by and maintenance of CD4 effector memory and effector T cell subsets in persistent malaria infection

**DOI:** 10.1371/journal.ppat.1006960

**Published:** 2018-04-09

**Authors:** Michael M. Opata, Samad A. Ibitokou, Victor H. Carpio, Karis M. Marshall, Brian E. Dillon, Jordan C. Carl, Kyle D. Wilson, Christine M. Arcari, Robin Stephens

**Affiliations:** 1 Departments of Internal Medicine, Division of Infectious Diseases, University of Texas Medical Branch, Galveston, TX, United States of America; 2 Department of Microbiology and Immunology, University of Texas Medical Branch, Galveston, TX, United States of America; 3 Department of Preventive Medicine & Community Health, University of Texas Medical Branch Galveston, TX, United States of America; University of Edinburgh, UNITED KINGDOM

## Abstract

Protection at the peak of *Plasmodium chabaudi* blood-stage malaria infection is provided by CD4 T cells. We have shown that an increase in Th1 cells also correlates with protection during the persistent phase of malaria; however, it is unclear how these T cells are maintained. Persistent malaria infection promotes protection and generates both effector T cells (Teff), and effector memory T cells (Tem). We have previously defined new CD4 Teff (IL-7Rα^-^) subsets from Early (Teff^Early^, CD62L^hi^CD27^+^) to Late (Teff^Late^, CD62L^lo^CD27^-^) activation states. Here, we tested these effector and memory T cell subsets for their ability to survive and protect *in vivo*. We found that both polyclonal and *P*. *chabaudi* Merozoite Surface Protein-1 (MSP-1)-specific B5 TCR transgenic Tem survive better than Teff. Surprisingly, as Tem are associated with antigen persistence, Tem survive well even after clearance of infection. As previously shown during T cell contraction, Teff^Early^, which can generate Tem, also survive better than other Teff subsets in uninfected recipients. Two other Tem survival mechanisms identified here are that low-level chronic infection promotes Tem both by driving their proliferation, and by programming production of Tem from Tcm. Protective CD4 T cell phenotypes have not been precisely determined in malaria, or other persistent infections. Therefore, we tested purified memory (Tmem) and Teff subsets in protection from peak pathology and parasitemia in immunocompromised recipient mice. Strikingly, among Tmem (IL-7Rα^hi^) subsets, only Tem^Late^ (CD62L^lo^CD27^-^) reduced peak parasitemia (19%), though the dominant memory subset is Tem^Early^, which is not protective. In contrast, all Teff subsets reduced peak parasitemia by more than half, and mature Teff can generate Tem, though less. In summary, we have elucidated four mechanisms of Tem maintenance, and identified two long-lived T cell subsets (Tem^Late^, Teff^Early^) that may represent correlates of protection or a target for longer-lived vaccine-induced protection against malaria blood-stages.

## Author summary

Malaria causes significant mortality but current vaccine candidates have poor efficacy and duration, as does natural immunity to malaria. T helper cells (CD4^+^) are essential to protection from malaria, but it is unknown what kinds of T cells would be both protective and long-lasting. Here, we explored the mechanisms of survival used by memory T cells in malaria, and their ability to protect immunodeficient animals from malaria. We identified four mechanisms by which memory T cells are maintained in chronic infection. We also showed that highly activated effector T cells protect better than memory T cells in general, however, effector T cells have a shorter lifespan suggesting a mechanism for short-lived immunity. In total, we identified two protective T cell subsets that are long-lived. Unfortunately, the memory T cell subset that protects, is not the predominant memory T cell population generated by natural infection, suggesting a mechanism for the poor immunity seen in malaria. Our work suggests that vaccines that induce these two T cell subsets may improve on current immunity from malaria infection and disease.

## Introduction

Malaria accounts for an estimated 438,000 deaths annually, with over 3 billion people at risk of infection [1]. *Plasmodium* infection can be considered chronic both for the repetitious exposure in hyperendemic areas [2], as well as for the ability of both *P*. *falciparum* and *P*. *vivax* infections to persist for years even in the absence of parasite transmission [3, 4]. *P*. *chabaudi* infection lasts up to 90 days in mice [5], making it a unique and well-accepted model to study the chronic phase of malaria infection. CD4 T cells play a central role in protection of chronic infections such as malaria, LCMV and *Leishmania* in mice, but the protection established wanes on cure of the infection. In *P*. *chabaudi* infection, complete protection from secondary parasitemia decays by 200 days post-infection [6]. This is accompanied by a decay in proliferation of CD4 T cells in response to parasite antigens *in vitro*, but not a decay in antibody titers, suggesting that T cell function mediates decay in protection. Chronic infection has also been shown to improve T cell-mediated protection [5–7]. Protection by the RTS,S vaccine, which will likely be implemented in some countries soon, varies from 12 to 68%, depending on the context and what outcomes are measured [8]. Although many malaria cases are likely to be averted with this first vaccine, total decay of the efficacy of the RTS,S vaccine occurs in just four years [9]. Even with the newer whole-parasite vaccines, which have higher reported efficacy, the phenotype of the T cells generated suggests that they may also be short-lived effector T cells [10]. Strategies that include treatment of infection with anti-malarial drugs may generate more long-lived T cell responses [11].

The specific CD4 T cell population observed after *P*. *chabaudi* infection is comprised of a mixture of effector (Teff) and memory (Tmem) phenotype T cells [7]. We showed that specific T cells in the memory phase do not re-expand in response to a second *P*. *chabaudi* infection [12]. While this could be explained by either Teff or Tem, it has been experimentally challenging to distinguish the phenotype of these two populations. In a recent elegant study, protective Teff in *Leishmania* infection were identified as proliferating, terminally differentiated cells expressing effector molecules, while effector memory T cells (Tem) were defined only at later timepoints as memory T cells expressing migration markers and effector molecules [13]. In our work, we have used the observation that IL-7Rα is completely but transiently downregulated on effector T cells, and re-upregulated on Tem, to distinguish two different populations with unique survival and protection capacity [7, 12, 14]. Tem do not undergo homeostatic proliferation [15], suggesting that they may not survive as long as Tcm in the absence of antigen. However, the actual survival potential of Tem is unknown, as studies to date do not distinguish Tem from Teff [16]. Even less is known about mechanisms of survival of Teff and Tem cells in chronic infections.

We have started to narrow down the characteristics of T cells that are most protective in malaria. Using adoptive transfer of MSP1-specific TCR Transgenic CD4 T cells to study the effect of chronic *P*. *chabaudi* infection on protection, we showed that the memory population (CD4^+^CD44^hi^CD25^-^) of previously activated T cells from chronically infected mice protected better than T cells from mice that were treated with chloroquine, an anti-malarial drug previously shown to clear *P*. *chabaudi* (AS), one month after infection [7]. We observed that the T cell population from chronically infected animals at the memory timepoint contained more Teff (CD127^-^), and more IFN-γ^+^TNF^+^IL-2^-^ cytokine producing T cells than those from treated animals. These Th1 cells were CD44^hi^ CD62L^lo^, indicating that they are either Teff or Tem maintained by chronic infection. More recently, we showed that most *Ifng*+ Teff do not maintain *Ifng* expression to the memory timepoint. However, all *Ifng*^+^ T-bet^+^ T cells derived from *Ifng*+ Teff that survive until d60, proliferate extensively between the peak of infection and the memory timepoint [14]. Collectively, these data suggest that maintenance of potentially protective Th1 cytokine production and Teff/Tem cells themselves is linked to persistent infection.

In order to define the precursors of Tem, we previously identified three subsets of Teff (CD127-) representing different stages of activation which are generated sequentially in *P*. *chabaudi* infection [12]. By day 5 post-infection (p.i.), early effector T cells (Teff^Early^, CD62L^hi^CD27^+^) are detectable, as they have down-regulated IL-7Rα/CD127, but have not yet lost CD62L expression. Strikingly, though some Teff^Early^ express IFN-γ, the majority of CD127^**-**^ Teff^Early^ have not expressed CD11a or proliferated. Teff^Early^ still have not proliferated, even on day 9 p.i., when T cell numbers peak in this infection. The majority of Teff at day 7 p.i. are CD62L^lo^CD27^+^ intermediate effector T cells (Teff^Int^), which are the first proliferating subset. Teff^Int^ express PD-1^int^ and high levels of Th1 cytokines. Finally, the majority of Teff lose both CD62L and CD27 expression, and become Teff^Late^ (CD62L^lo^CD27^-^) by day 9 p.i. Teff^Late^ have high levels of phosphatidyl serine in their outer plasma membrane leaflet, indicating susceptibility to apoptosis, as previously reported for CD27^**-**^ CD8 T cells [17]. All three Teff subsets contain cells expressing the Th1- cytokine, IFN-γ, and transcription factor, T-bet. Notably, upon transfer into recipients at the peak of infection, Teff^Early^ survive the T cell contraction phase better than the intermediate and late Teff subsets [12]. The increased survival of Teff^Early^ is supported by their higher transcription of the pro-survival genes Bcl2, Mcl1, Pim2, and Pim3. These anti-apoptotic molecules are down-regulated concomitant with CD62L down-regulation, which also signals terminal differentiation and expression of PD-1 and Fas. Identification of these Teff subsets allowed purification of activation intermediates and facilitates the study of effector and memory T cell differentiation and maintenance *in vivo*.

To determine the pathway of differentiation of effector memory T cells (Tem) in chronic infection, we used these three Teff subsets in adoptive transfer experiments in *P*. *chabaudi* infection [18]. We found that Teff^Early^ can generate central memory T cells (Tcm) in uninfected recipients. We have shown in the past that Tcm, in turn, can generate Tem during the high-level chronic infection of RAG2^o^ animals without B cell transfer [7, 12]. In contrast, Teff^Late^, which share the CD62^lo^CD27^-^ phenotype, are generally short-lived, though they have the plasticity to survive and expand highly in infected RAG2^o^ animals. Therefore, our data supports a model where Tem are generated from Tcm, not Teff, as often proposed, suggesting the possibility of a longer lifespan [19].

In the current study, we purified Tmem and Teff subsets from Merozoite Surface Protein-1 (MSP-1)-specific T cell receptor transgenic (B5 TCR Tg) mice, and tested their ability to survive in uninfected and/or chronically-infected recipients. We also tested their ability to protect immunodeficient animals in coordination with B cells. In the course of this work, we observed four mechanisms for promoting Tem survival in chronic infection. These mechanisms are: 1) Tem can survive in the absence of antigen; 2) some Teff may survive to generate Tem, as Teff are capable of re-upregulating CD127 over time, and remain protective; 3) Tcm derived from *P*. *chabaudi*-infected animals continue to generate Tem, even after infection is cleared; 4) low-level persistent infection promotes proliferation of Tem. On testing the Teff and Tmem for protection of immunodeficient animals from malaria, we show that among Tmem subsets, only Late effector memory T cells (Tem^Late^, CD127^hi^ CD62L^lo^ CD27^-^) reduce both pathology and parasitemia slightly. In contrast, all Teff subsets strongly reduce parasitemia, though they lose this ability over time. Interestingly, Teff^Early^ both protect well and survive in these assays, while other Teff are shorter-lived. These results have the potential to both explain poor, short-lived protection from malaria and to inform novel methods to drive long-lived protection by vaccination.

## Results

### Effector memory T cells do not decay upon clearance of infection

**Fig 1:**
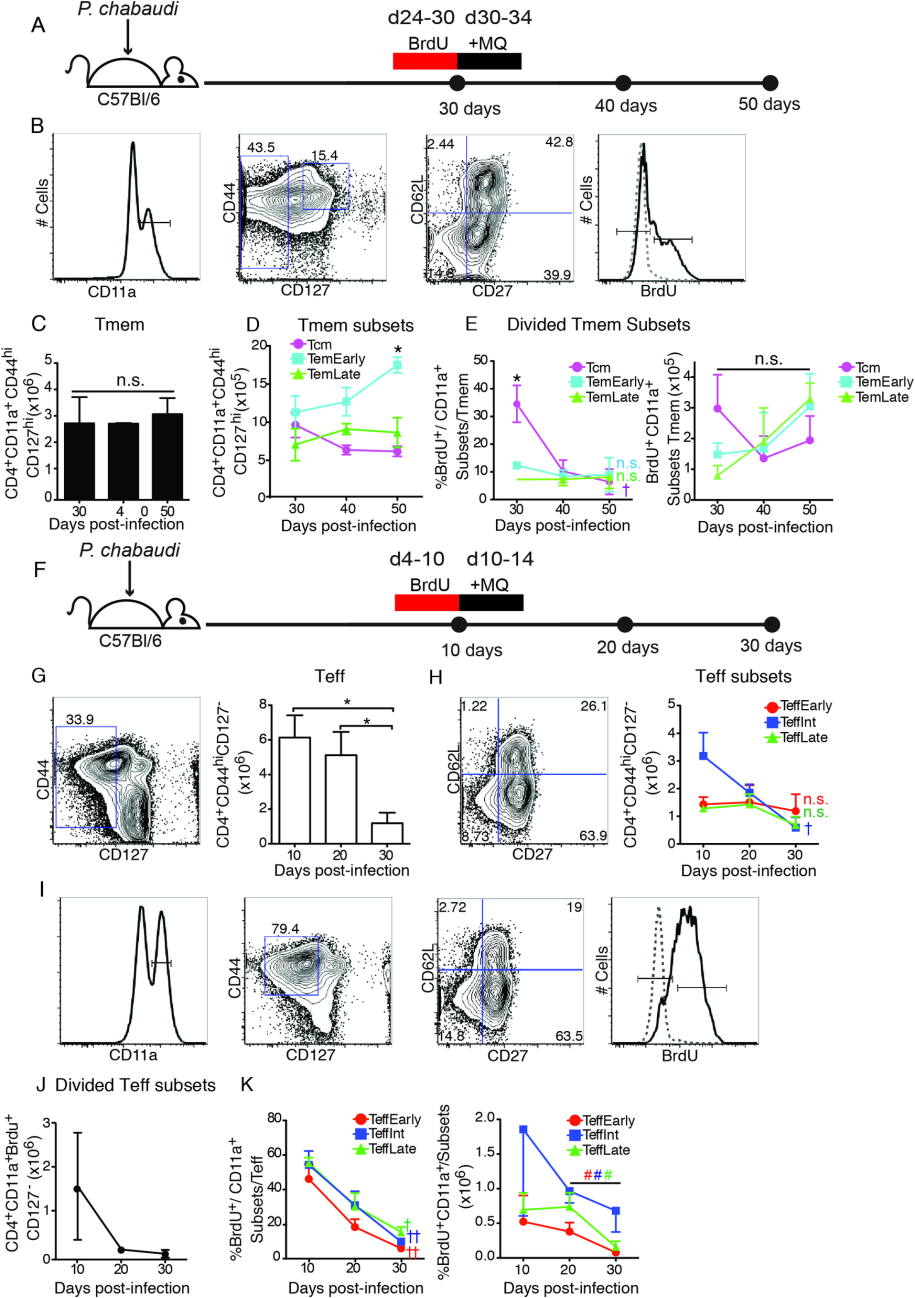
Mature effector T cells decay faster than effector memory T cells in *P*. *chabaudi*. Decay of malaria-specific polyclonal cells was detected by infecting C57Bl/6 mice with *P*. *chabaudi* and administration of BrdU either (**A-E**) during the memory phase (days 24–30), or (**F-K**) the peak of infection (days 4–10) to label Teff. The day after the end of BrdU administration, the infection was terminated with mefloquine (MQ), and decay of CD4^+^ memory (CD44^hi^ CD127^hi^ CD11a^+^, BrdU^+/-^) and effector (CD127^**-**^, (CD11a, BrdU)^+/-^) T cells in the spleen was determined by flow cytometry. **A)** Schematic representation of the experimental design for memory phase. **B)** Plots show the gating strategy for Tmem, including BrdU. Graphs showing **C)** number of Tmem (CD4^+^ CD11a^+^ CD44^hi^ CD127^hi^) and **D)** survival of memory T cell subsets in the spleen after parasite clearance. **E)** Graphs showing percentage (left) and number (right) of cells in each Tmem subset that survive after proliferating days 24–30 (BrdU^+^). **F)** Schematic representation of the experimental design to study decay of effector T cells, where BrdU was given days 4–10 p.i., and infection was terminated with MQ days 10–14 p.i. Flow cytometric gating and graphs showing **G)** number of Teff (CD4^+^ CD127^**-**^) and **H)** survival of Teff subset populations in the spleen after parasite clearance. **I)** Plots showing the gating strategy for Teff from CD11a+ to Teff (CD4^+^ CD11a^+^ CD44^hi^ CD127^lo^). Graphs showing **J)** the number of divided Teff (CD4^+^ CD11a^+^ CD127^-^ BrdU^+^), as they decay after labelling days 4–10 p.i, and subsequent parasite clearance, and **K)** the percentage (left) and number (right) of cells in each of the divided Teff subsets as they decay. Data represent 3 mice per group. Data was analyzed by Student’s *t* test and error bars represent SEM. * represents a significant difference between subsets at one timepoint. † represents a significant difference between timepoints d10-d30, or d30-50, # from d20-30 only; one symbol p<0.05, two symbols p<0.01, n.s.–not significant with color coding of symbols to indicate which subset changes.

While the current paradigm holds that Tmem survive longer than Teff, there is little data in the literature on the relative lifespans of Tem and Teff, particularly after clearance of antigen or pathogen. Therefore, we investigated the decay of polyclonal T cells responding to *P*. *chabaudi* infection upon clearance of infection. We tracked potential decay of three memory T cell (CD127^hi^) subsets (Tcm, CD62L^hi^CD27^+^; Tem^Early^, CD62L^lo^CD27^+^, Tem^Late^, CD62L^lo^CD27^-^), and three effector T cell (CD127^**-**^) subsets (Teff^Early^, CD62L^hi^CD27^+^; Teff^Int^, CD62L^lo^CD27^+^; Teff^Late^, CD62L^lo^CD27^-^) after treatment of infection, as shown schematically in **Fig 1A–1F**. Infection was stopped by day 34 using the anti-malarial drug, mefloquine (MQ). We gated on CD11a^+^ cells and determined memory (CD44^hi^CD127^hi^), Tmem subsets, and proliferation using BrdU **(Fig 1B)**, then quantified the number and proportion of proliferating Tmem (CD4^+^ CD44^hi^ CD127^hi^ CD11a+, **Fig 1C and 1D**) in the spleen of C57BL/6 animals using CD11a, as it was recently described to be upregulated only on MHC/peptide-stimulated T cells, and not on T cells activated by cytokines [20]. We have studied the lymph nodes in this infection, and they contain few T cells responsive to malaria, and equal ratios of Tcm and Tem at memory timepoints [7]. We quantified the number of polyclonal Tcm, Tem^Early^, and Tem^Late^ over a 30-day period by flow cytometry, after clearance of parasite in the spleen with MQ treatment (**Fig 1E**). We found relatively stable numbers of all Tmem subsets. In addition, we tracked the survival of Tmem after the clearance of parasite by labelling Tmem that had proliferated late in infection and then administering anti-malarial drug. These Tmem may have proliferated specifically in response to the low levels of parasite, or homeostatically, which would increase Tcm labelling. To quantify proliferation, we administered 5-Bromodeoxyuridine (BrdU), which is incorporated into the DNA of proliferating cells, on days 24–30 p.i., just before anti-malarial treatment. Strikingly, the proportions of BrdU^+^ Tem measured over the 20-day period did not decay (**Fig 1E, left**). The percent of BrdU^+^ Tcm out of Tmem does decay initially; however, none of the Tmem subsets decay significantly in cell number over this time period (**Fig 1E, right**).

We also tracked the survival of polyclonal Teff (IL-7Rα/CD127^**-**^) after clearance of parasite by treating *P*. *chabaudi* infected mice with mefloquine on days 10–14 p.i **(Fig 1F)**. The number of polyclonal effector T cells (CD4^+^ CD127^-^) in the spleen of mefloquine-treated mice decayed significantly in the 20 days post-treatment (day 30 p.i., **Fig 1G**), suggesting an intermediate lifespan. The decay in number of the largest Teff subset, Teff^Int^ (CD62L^lo^ CD27^+^), is easily seen, while Teff^Early^ (CD62L^hi^ CD27^+^) remained stable (**Fig 1H**), showing strong survival as we previously showed in the T cell contraction phase from days 8–11 p.i. [12]. The number of Teff^Late^, the most terminally differentiated subset, also remains stable. This stability likely represents Teff^Int^ transition to the Teff^Late^ population and then die [12]. Similar observations have also been suggested for CD8 CD27^**-**^ [17].

When we gated on CD11a^+^ T cells that had divided in response to infection (BrdU^+^), the decline of Teff numbers was variable, but rapid (**Fig 1I and 1J**). This decline was similar in the proportions and numbers of all the BrdU^+^ Teff subsets **(Fig 1K)**. The difference between the decay in the number of Teff^Early^ by day 30 when gated on divided cells (CD127^-^CD11a^+^ BrdU^+^), and the better maintenance of undivided (BrdU^**-**^) Teff^Early^ is interesting. This difference suggests that while the Teff^Early^ population survives better than other Teff subsets [12], there is a fraction of Teff^Early^ that are CD11a^+^ and proliferate, and then decay like the other proliferative Teff subsets. Taken together, this data suggests that Teff generated in malaria infection decay over 20 days in the absence of parasite, while Tmem, including Tem, are more stable and long-lived.

**Fig 2:**
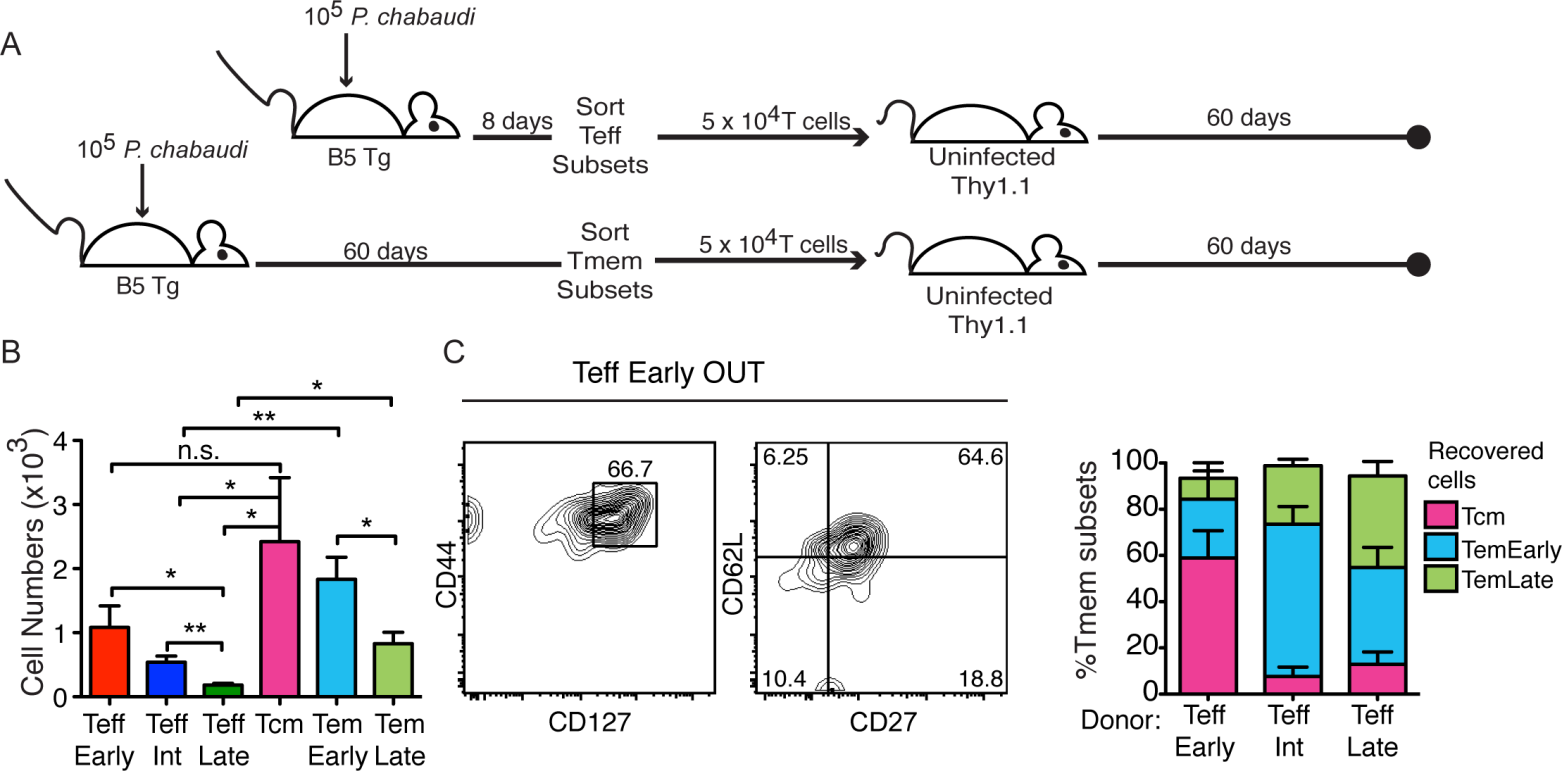
Teff^Early^ survive like Tmem cells, while highly activated Teff subsets decay. **A)** Schematic representation of the experimental design. T cell subsets were sorted from spleens of infected B5 TCR Tg animals (Effector on d8 p.i. (top) and Memory on d60 p.i. (bottom)) and the same number of T cells of each subset (5 x 10^4^) were transferred into uninfected congenic recipients (Thy1.1) for 60 days. **B)** Graph showing numbers of B5 TCR Tg (CD4^+^ Thy1.2^+^) T cells recovered from spleens of recipients of each T cell subset on d60 post-transfer. Data represent 4–9 mice per group from two experiments. Data were analyzed using Student’s *t* test, **p<0.01, *p<0.05. n.s.–not significant. **C)** Concatenated contour plot of B5 T cells recovered from Teff^Early^ recipients day 60 post-transfer showing memory (CD44, CD127) and memory T cell subset (CD62L, CD27) phenotype, and summary graph of Tmem phenotype of recovered T cells from the groups of mice that received each Teff subset. Error bars represent SEM.

Teff and Tmem survival cannot be understood only from studying the polyclonal response due to the changing phenotypes of activated T cells, particularly the re-upregulation of CD127 on Teff. Therefore, we tested the ability of highly-purified subsets of MSP1-specific B5 TCR Tg T cells to survive in uninfected Thy1.1 congenic recipients after adoptive transfer of a physiological number (5 x 10^4^). T cells from infected B5 Tg donors of each Teff (CD127^**-**^, d8 p.i.) and Tmem (CD44^hi^ CD127^hi^, d60 p.i.) subset were transferred into groups of uninfected congenic (Thy1.1) recipients, as shown schematically in **Fig 2A**. Donors were age matched, and Teff and Tmem sort was completed on the same day so that recipient flow cytometry could be performed on the same day. Recovered B5 TCR Tg T cells (Thy1.2^+^ CD4^+^) were counted after two months (**Fig 2B**). We observed significantly more Teff^Early^ cells than terminally differentiated Teff^Late^ that survived for two months. These results confirm our previous studies which demonstrated a clear survival advantage for Teff^Early^ compared to other Teff subsets over two weeks [12]. Recipients of both Tem subsets had significantly higher numbers of Thy1.2^+^ T cells by day 60 post-transfer than recipients of CD127^**-**^ Teff cells with similar CD62L and CD27 phenotypes, Teff^Int^ and Teff^Late^. We concluded that survival was more robust for all of the memory T cell subsets, including Tem, compared to survival of the terminal Teff. Importantly, survival of Teff^Early^ (CD62L^hi^ CD27^+^, CD127^**-**^) was not significantly different than survival of Tcm (CD62L^hi^ CD27^+^, CD127^hi^), consistent with our previous studies suggesting that Tcm and Teff^Early^ are closely related [12]. The surviving Teff^Early^ population re-upregulated CD127 and became largely CD127^hi^ in uninfected Thy1.1 recipients over two months (**Fig 2C**). CD127 re-upregulation starts on day 14 post-transfer, as we previously reported [12]. While transition to CD127^hi^ is not definitive evidence of a memory phenotype, Teff^Early^ do survive in similar numbers as Tcm, suggesting that surviving Teff^Early^ cells become Tcm, and that the few surviving mature Teff can become Tem. Taken together, these experiments suggest that Tem survive in the absence of chronic infection, and that even in malaria infection, where immunity decays, memory T cells are longer-lived than terminally differentiated effector T cells.

### Mechanisms of maintenance of effector memory T cells by chronic infection

**Fig 3:**
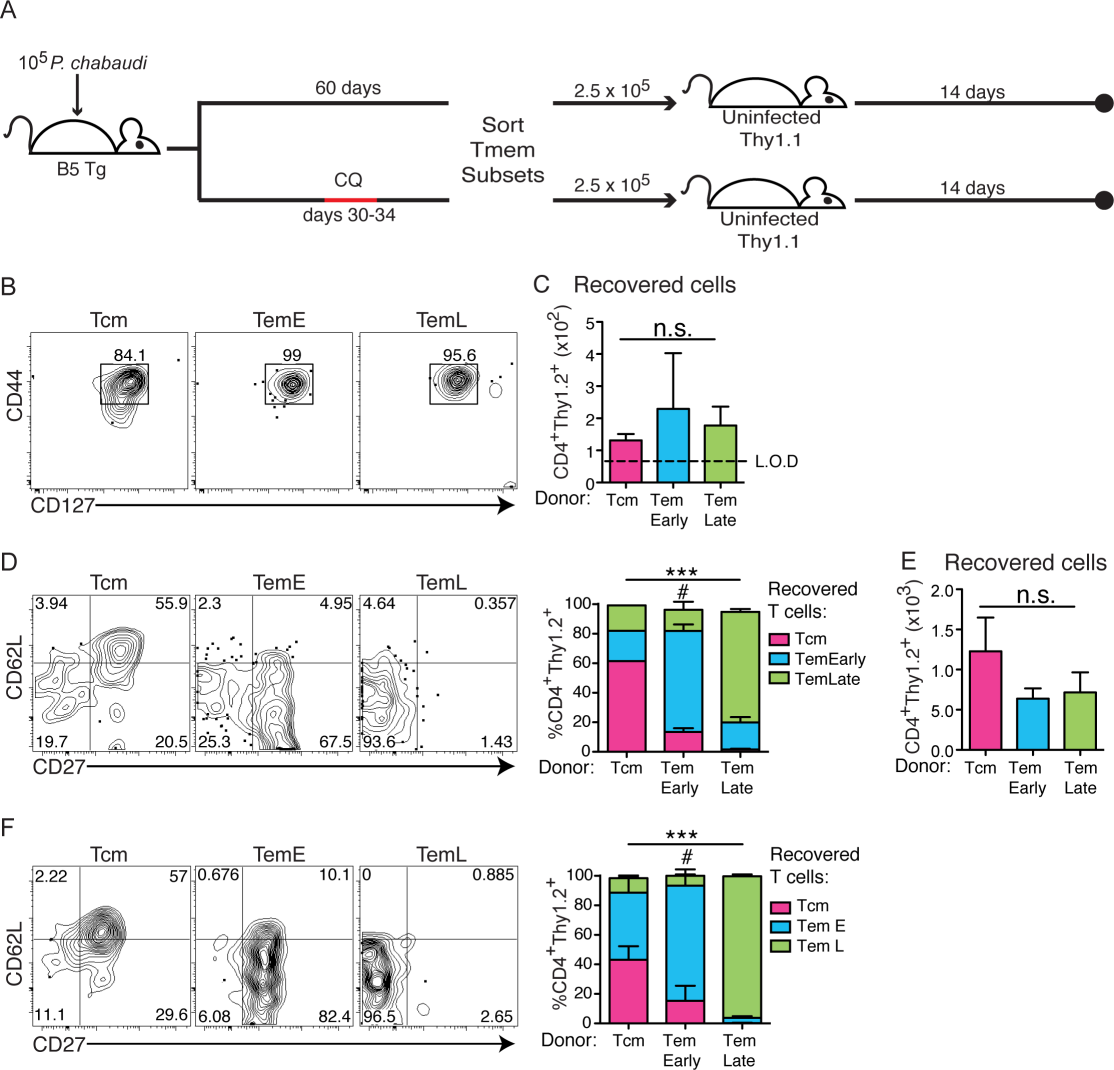
CD4 Memory T cell subsets differentiate from Tcm to Tem^Late^ even after elimination of chronic infection. **A)** Schematic representation of the experimental design. (**top**) Memory T cell subsets from infected B5 TCR Tg donors (Thy1.2), that were not treated (**A-D**), or had been treated (**bottom, E, F**) with chloroquine (CQ) on days 30–34, were sorted from the spleen (d60 p.i.), and transferred (2.5 x 10^5^) into uninfected congenic (Thy1.1) recipients for 14 days. **B)** Concatenated contour plots with outliers showing the memory phenotype (CD44, CD127) of all B5 T cells (CD4^+^ Thy1.2^+^) recovered from all animals in each group. **C)** Numbers of B5 T cells recovered from spleens of recipients of Tmem from untreated donors post-transfer. **D)** Concatenated contour plots and summary stacked bar graph showing memory subset phenotypes (CD62L, CD27) of all B5 memory T cells recovered from all animals in each group. Summary bar graph shows average in each Tmem subset gate on recovery. (**E, F**) Memory T cell subsets sorted from infected mice treated with anti-malarial drug, CQ, were recovered from uninfected recipients. **E)** Numbers of B5 T cells recovered from recipients of B5 Tmem from CQ treated donors are shown. **F)** Concatenated contour plots and summary graph with outliers showing subset phenotypes of B5 Tmem recovered from recipients of B5 Tmem from CQ treated donors. Summary bar graph shows average in each Tmem subset gate on recovery. Data represent 2–3 mice per group. Error bars represent SEM, and n.s–no significant difference between all groups, ***p<0.001 comparing the distribution of the subset phenotypes of B5 T cells recovered in the three groups of recipients, ^**#**^p<0.05 comparing the fraction of chronically stimulated vs. rested Tem^Early^ donor cells recovered as Tem^Late^ in panels D and F.

Durability of memory T cells is a critical feature for their function. However, there is relatively little that is known about the ability of CD4 Tem to survive in the absence of antigen [16, 21]. Therefore, we tested the phenotype of surviving Tmem generated in this infection after transfer and competition with endogenous T cells in congenic mice. Memory T cell subsets were sorted from B5 TCR Tg animals infected two months earlier, using the gating strategy shown in **S1A Fig.** These highly-purified populations were generated from untreated B5 TCR Tg donors, or donors treated with chloroquine (CQ) on days 30–34 p.i. to eliminate persistent parasite. Each of three sorted Tmem subsets were transferred (2.5 x 10^5^) into a group of uninfected Thy1.1 recipients, as shown schematically in **Fig 3A**. B5 TCR Tg T cells (Thy1.2^+^ CD4^+^) were recovered and analyzed by flow cytometry after two weeks. On recovery, all Tmem subsets maintained their original memory phenotype (CD44^hi^CD127^hi^, **Fig 3B**). The number of T cells recovered after two weeks from each group of recipients was similar (**Fig 3C**), and the number of cells in each recipient was clearly detectable above the limit of detection (L.O.D.) in all animals. Interestingly, the T cells recovered from recipients of Tcm had progressed to include some Tem^Early^ and Tem^Late^ phenotype cells (**Fig 3D**), while the Tem^Early^ recipients also generated Tem^Late^ cells in some animals (3/5), potentially reflecting the individual variation in time to clearance of parasite. The majority of Tem^Late^ maintained their original phenotype. We previously described this differentiation pathway in the context of a chronic infection with high parasitemia in immunodeficient RAG2^o^ animals [7], but it was unexpected to see this progression in immunocompetent and T cell replete recipient animals, especially in the absence of parasite antigen in the recipient.

We hypothesized that the continued differentiation of memory T cell subsets from Tcm to Tem in the uninfected recipient animals could be due to T cell “programming” in the context of chronic infection, which can last up to day 90 p.i., in donor animals. Therefore, to test if progressive differentiation of memory T cells was due to continuous exposure to infection, Tmem donor mice were infected with *P*. *chabaudi* (1 x 10^5^ iRBCs), and then treated with chloroquine (CQ) starting at day 30. Chloroquine completely clears low levels of parasitemia in *P*. *chabaudi* (AS) infection [5, 22], therefore, parasite is eliminated in +CQ donors for the month prior to Tmem sorting and transfer. Memory T cell subsets were sorted on d60, 26 days after final chloroquine treatment of the donor, and they were transferred into uninfected Thy1.1 hosts. Similar numbers of T cells were recovered 14 days post-transfer regardless of the chloroquine treatment of the donor animals (**Fig 3E**). While the pattern of Tmem subsets of B5 T cells recovered from Tcm transfer was similar after chloroquine treatment of donors, there was a significant reduction in progression of Tem^Early^ to Tem^Late^ when Tem^Early^ had not been exposed to parasite for several weeks before transfer (**Fig 3F,** p = 0.0499), with rested (+CQ) cells recovered from all animals (4/4) exhibiting little progression to Tem^Late^. These data indicate that Tcm cells in this infection can continue to progress towards more highly differentiated Tmem subsets for some time after clearance of parasite. However, in the recent absence of persistent infection, the Tem^Early^ do not make Tem^Late^.

**Fig 4:**
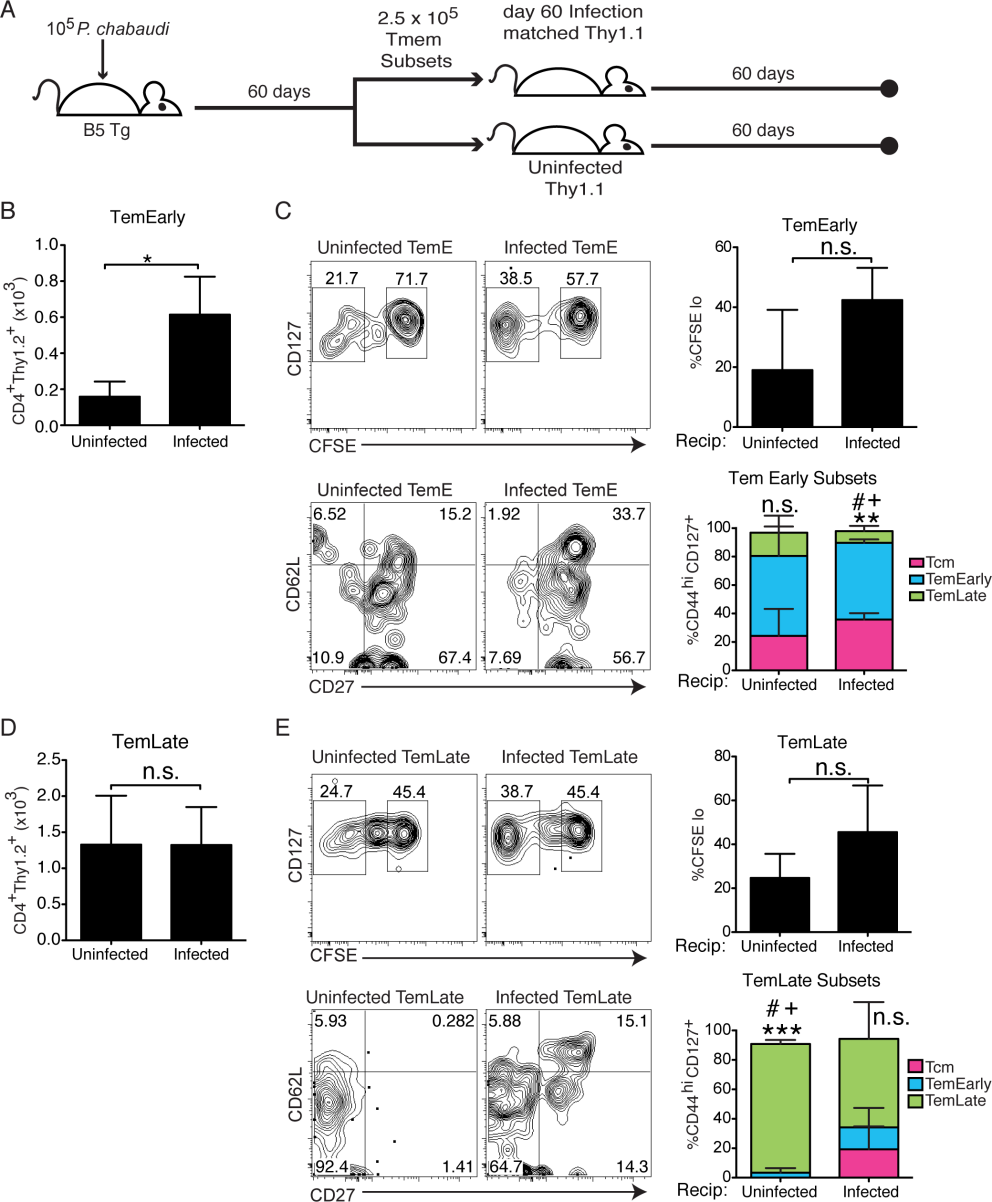
Persistent infection promotes Tem survival. **A)** Schematic representation of the experimental design. Memory T cell subsets were sorted from infected B5 TCR Tg mice (d60 p.i.) and the same number of each subset (2.5 x 10^5^) were transferred into either infection-matched (d60 p.i., top) or uninfected (bottom) Thy1.1 hosts for 60 days after transfer. **B)** Graph shows numbers of recovered B5 T cells (CD4^+^ Thy1.2^+^) in recipients of Tem^Early^. **C)** Concatenated contour plots show division (CFSE-), and levels of CD127, and phenotypes (CD62L, CD27) of cells recovered from Tem^Early^ recipients. Summary bar graphs show average of divided cells or fraction in each Tmem subset gate on recovery. **D)** Graph shows numbers of recovered B5 T cells in recipients of Tem^Late^. **E)** Concatenated contour plots show levels of CFSE, and CD127, and phenotypes (CD62L, CD27) of cells recovered from Tem^Late^ recipients. Summary bar graphs show average of divided cells or fraction in each Tmem subset gate on recovery. Data are representative of 2–4 mice per group from 2 similar experiments. Data was analyzed by Student’s *t* test, and error bar represents SEM, *p<0.05, n.s.–not significant. On summary bar graphs symbols refer to differences between fractions of recovered cells in each Tmem subset in a given stacked bar, one symbol = p<0.05; two symbols, **p<0.01 comparing Tcm to TemL; three symbols, ***p<0.001 comparing Tem^Early^ to Tem^Late^, ^***#***^ comparing Tcm (very few) to Tem^Early^, ^**+**^ comparing Tem^Early^ to Tem^Late^.

Tem are generally found associated with chronic infection [23, 24]; however, we observed similar survival of Tem and Tcm even in the absence of antigen. Therefore, we tested if transferring Tem into the environment of low-level chronic infection would improve Tem numbers compared to uninfected hosts. Tem subsets were sorted from infected (d60 p.i.) B5 TCR Tg mice, and transferred into infection-matched (d60 p.i.) Thy1.1 recipients with sub-patent (below-detectable) parasitemia, or uninfected hosts, as shown schematically in **Fig 4A**. The number of B5 T cells recovered from infection-matched recipients after two months compared to uninfected recipients was significantly higher in Tem^Early^ recipients (**Fig 4B**). Interestingly, most B5 T cells recovered from Tem^Early^ recipients maintained their high level of CD127 expression **(Fig 4C)**, even in the presence of sub-patent levels of parasite. T cells from all infection-matched recipients showed a distinct peak of divided T cells (CFSE^**-**^); however, this difference (%CFSE- Tem^Early^ infected and uninfected recipients) did not reach statistical significance. The majority of Tem^Early^ maintained their phenotype; however, some of the recovered cells progressed, or re-upregulated CD62L. Tem^Late^ do not accumulate in low-level chronic infection. Similar to Tem^Early^, Tem^Late^ from infection-matched recipients showed a distinct peak of divided T cells, however, this difference (%CFSE- Tem^Early^ infected and uninfected recipients) did not reach statistical significance (**Fig 4E,** top panels). The Tem^Late^ recovered from uninfected recipients retained their original phenotype remarkably well in all recipients (**Fig 4E,** bottom panels). Therefore, it appears that Tem survive similarly to Tcm in the absence of infection, but Tem have additional mechanisms to promote their longevity in chronic infection accounting for their increased fraction compared to Tcm.

### Of the three memory T cell subsets, only late effector memory T cells protect immunodeficient animals from malaria infection

**Fig 5:**
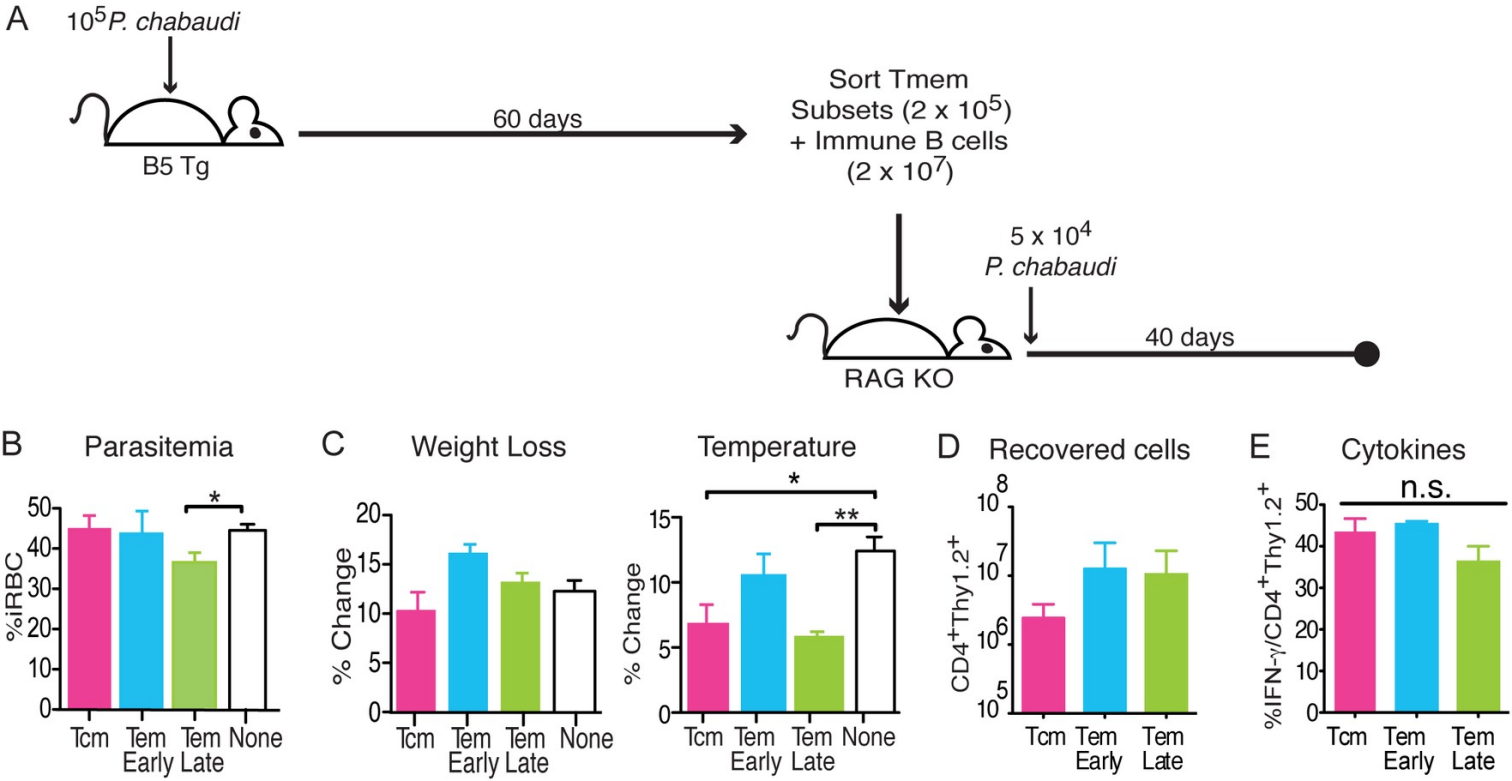
Late effector memory T cells protect immunodeficient animals from malaria. **A)** Schematic of the experimental design. Memory T cell subsets were sorted from spleens of infected B5 TCR Tg mice on d60 p.i, and transferred (2 x 10^5^) with immune B cells (2 x 10^7^) into RAG2^o^ mice. Recipient mice were then infected with *P*. *chabaudi* (5x10^4^ iRBC). Parasitemia, weight, and temperature were assessed daily for 40 days p.i., and splenocytes were analyzed by flow cytometry on day 40. Graphs of the average **B)** peak parasitemia, and percent change of **C)** weight and hypothermia of recipient mice at the peak of each symptom for each recipient (d8-10 p.i.). Flow cytometric analysis summarized here as **D)** total cell numbers of B5 T cells (CD4^+^Thy1.2^+^) recovered and **E)** the percent of B5 T cells that are IFN-γ^+^. Data shown represent 3 mice per group and are representative of 5 independent experiments. Error bar represents SEM, *p<0.05, **p<0.01, n.s.–not significant.

In the long-term, *P*. *chabaudi* infection primarily drives generation of effector memory T cells. In order to understand the potential role of Tem in protection from high parasitemia and pathology, we compared the effects of Tcm and Tem cells on survival, parasitemia, and pathology in infected immunocompromised mice. Using *P*. *chabaudi* infection of RAG animals to study the contribution of adaptive immunity to protection, we previously established that the peaks of parasitemia and pathology are only controlled by activated T cells; yet, the full clearance of parasite depends on high levels of antibody [7, 25]. As in the studies above, to test the potential of Tmem subsets to protect, we used subsets we have previously established in this model for memory T cells (CD127^hi^): Tcm (CD62L^hi^CD27^+^), Tem^Early^ (CD62L^lo^CD27^+^), and Tem^Late^ (CD62L^lo^CD27) [7]. The same number of B5 TCR Tg donor CD4^+^ T cells (2 x 10^5^) from each subset and immune BALB/c B cells (CD19^+^, 2 x 10^7^) were both transferred into groups of RAG2^o^ mice, which were then infected the following day with *P*. *chabaudi*, as shown schematically in **Fig 5A**. Infected RAG2^o^ mice that received B cells but no T cells were used as controls to measure changes induced by T cells at the peak of infection. Another appropriate control could have been naïve T cells, but we have previously shown that there is no significant difference in the peak parasitemia or pathology between infected RAG2^o^ mice with naïve T cells and B cells, or B cells alone, so we chose to use the no T cell control in each experiment as a universal control [7]. Parasitemia levels, weight loss, temperature, and cytokine levels were measured over the course of infection (**Fig 5B–5E**). We report an average of each animal’s maximal change, as each animal can exhibit peak parasitemia and pathology on a different day between days 8–10, as we have reported before [7]. Comparing the average peak parasitemia, Tem^Late^ was the only subset that significantly reduced parasitemia, compared to the universal control group that received immune B cells but no T cells (by an average of 8.7% iRBC/RBC, or 19% of peak RAG2^o^ parasitemia), while Tem^Early^ and Tcm did not reduce parasitemia (**Fig 5B**). Tem^Late^ and Tcm also significantly reduced peak hypothermia compared to control (**Fig 5C**). Transferred T cells expanded dramatically in all groups after 38 days of infection, as evidenced by the high numbers of recovered cells **(Fig 5D)**. Interestingly, this suggests similar abilities by all memory subsets to expand in the context of high parasitemia and leukopenia. Similar results were observed when cells were taken out on day 14 post-infection. While there were no differences in IFN-γ production by the T cells recovered from RAG recipients on day 38 (**Fig 5E**), we have previously shown that the three Tmem subsets transferred into congenic mice, instead of RAG2^o^ mice, exhibit strikingly different cytokine profiles. For instance, Tem^Late^ contain discreet IL-10+ and IFN-γ+ populations by two months after infection [7]. In summary, these data show that Tem^Late^ generated by chronic infection are able to contribute to reduction of peak parasitemia, though further study is required to determine the effector mechanisms responsible for the differences.

### All effector T cell subsets protect immunocompromised mice against malaria

**Fig 6:**
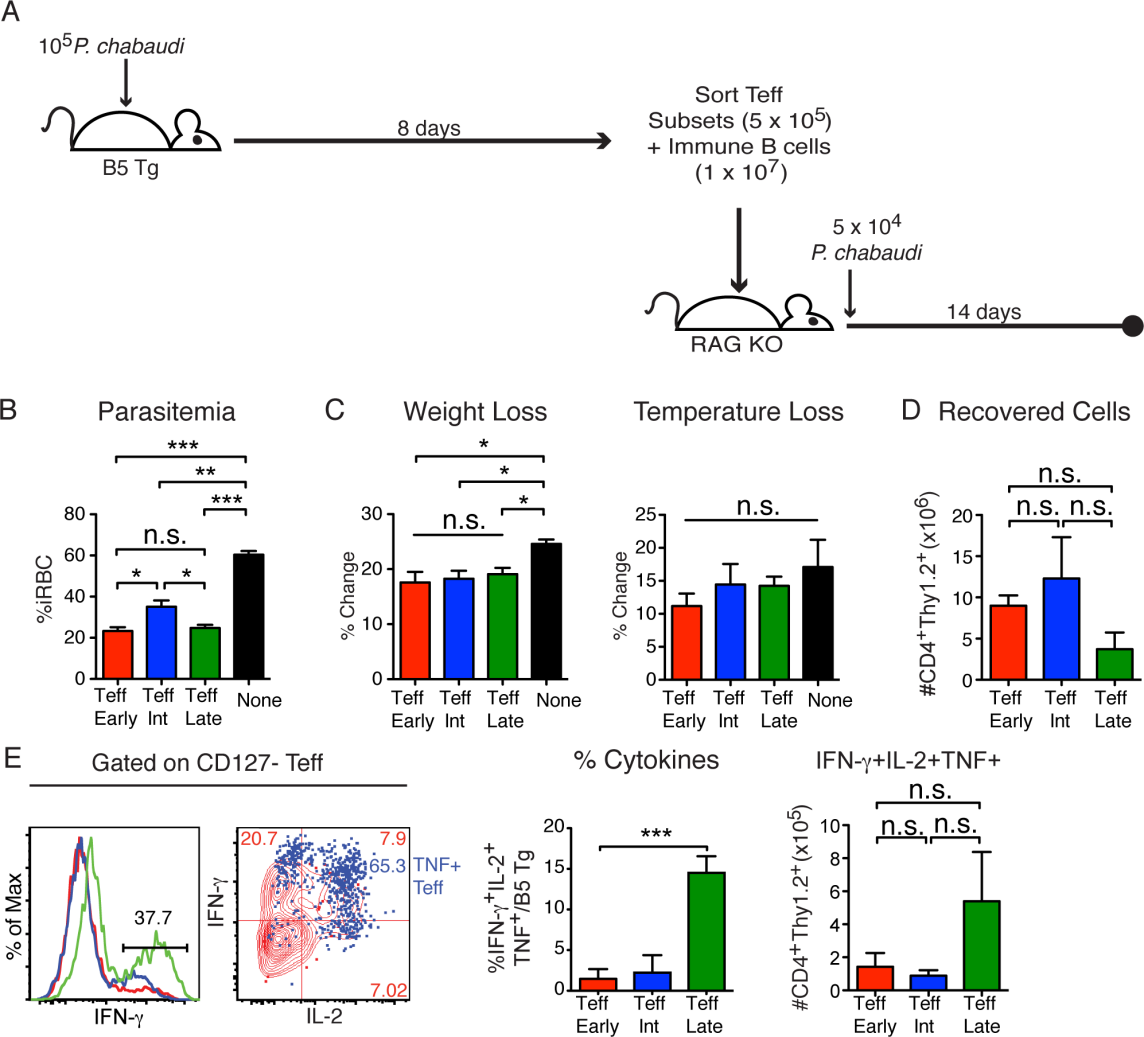
All Effector T cell subsets protect from parasitemia. **A)** Schematic of experimental model. Effector T cell subsets were sorted from spleens of B5 TCR Tg on d8 p.i., and transferred (5x10^5^) with immune CD19^+^ BALB/c B cells (1x10^7^) into RAG2^o^ mice that were then infected with *P*. *chabaudi* (5x10^4^ iRBC). Parasitemia and pathology were followed for two weeks. Graphs showing average **B)** peak parasitemia (%iRBC/RBC) summarized from two experiments (n = 6), **C)** % change of weight, and hypothermia of recipient mice at the peak of each symptom for each recipient (d8-10 p.i.). Flow cytometry of splenocytes was done on day 14 p.i. and **D)** graph shows average number of B5 T cells (CD4^+^Thy1.2^+^) recovered. **E)** Histograms, contour overlay (Teff^Late^), and summary graphs of cytokines produced by T cells from recipients of each Teff subset. Data show 2–3 mice per group and are representative of 3 independent experiments. Error bars show SEM, *p<0.05, **p<0.01, ***p<0.001, n.s–not significant.

Effector T cells are responsible for clearance of primary infection, but are thought to become terminally differentiated and die in the process. However, the lifespan of CD4 Teff populations during specific infections has rarely been determined. We sought to determine if the degree of maturation of the effector T cells affects their ability to provide protection from *P*. *chabaudi* infection. The CD127^-^ Teff ^Early^ (CD62L^hi^CD27^+^), Teff^Int^ (CD62L^lo^CD27^+^), and Teff^Late^ (CD62L^lo^CD27^-^) subsets were sorted from B5 TCR Tg donors on day 8 post-infection as shown with the gating strategy in **(S1B Fig**), and transferred (5x10^5^) into immunodeficient RAG2^o^ mice together with B cells (1x10^7^) from immune BALB/c mice, as in previous work [7, 25]. Recipient mice were infected with *P*. *chabaudi* one day post-transfer and parasitemia, weight loss, and hypothermia were monitored over 14 days, as shown schematically in **Fig 6A**. The average peak parasitemia showed that all of the effector T cell subsets significantly protected the recipient mice by an average of 28% iRBC/RBC, or 59% of peak RAG2^o^ parasitemia, compared to the control group (**Fig 6B**). Weight loss and hypothermia were measured at the peak of infection for each mouse (d8-10) as indicators of pathology. Weight loss, but not hypothermia, showed a significant difference between Teff groups and the control (**Fig 6C**). After 14 days of infection, all Teff populations were recovered in similar numbers (**Fig 6D**). Further, all the Teff subsets responded to the infection by producing cytokines on day 14 p.i (**Fig 6E**). The Teff^Late^ population produced higher proportions of all three cytokines (p = 0.0002). However, the number of triple-cytokine producers (TNF^+^IFN-γ^+^IL-2^+^) was similar in all recipient groups, suggesting a mechanism for the equal protection provided, as multi-cytokine producers correlate with protection in several infections [26].

As Teff protect so well, we next tested the possibility that Teff can be intermediate-lived and/or become memory T cells in the absence of exposure to parasite, and possibly contribute to protection. To this end, the three Teff subsets (Teff^Early^, Teff^Int^, Teff^Late^) were sorted and transferred into uninfected RAG2^o^ animals for two weeks, as shown schematically in the top panel of **S2A Fig**. As previously shown in wildtype congenic recipients [12], more Teff^Early^ cells than the mature Teff subsets were recovered after two weeks of “resting” (**S2B Fig**). Our previous study showed that the Teff^Early^ subset also contains precursors to memory T cells, while the Teff^Int^ and Teff^Late^ subsets decay [12]. Consistent with this observation, T cells recovered after 14 days from Teff^Early^ recipients showed re-upregulation of CD127 (**S2C Fig**). As we demonstrated previously in wildtype recipients, we observed that Teff^Early^ progressed along the pathway of differentiation and generated all of the Teff and Tmem subsets **(S2D Fig)**, while recipients of Teff^Int^ and Teff^Late^ had too few cells at this timepoint to determine their phenotypes. While the purpose of this experiment was to test protection, the potential of Teff^Early^ to make both Teff and Tmem subsets in this second model confirms the differentiation pathway largely defined in our previous work [12], and now summarized in our final figure.

To test the functional ability of Teff cells over time, we determined the relative ability of T cells surviving from each Teff subset to contribute to protection after 2 weeks in uninfected recipients **(S2A Fig, bottom)**. Teff subsets (Teff^Early^, Teff^Int^, Teff^Late^) were sorted from infected B5 TCR Tg donors on d8 p.i. and transferred into RAG2^o^ mice. These mice were then given B cells, and infected with *P*. *chabaudi* two weeks later. All Teff subsets still showed a trend of reducing peak parasitemia within 14 days, though this was only significant for Teff^Int^ (**S2E Fig**). Both Teff^Early^ and Teff^Int^ significantly protected recipients from weight loss, while Teff^Late^ significantly protected from hypothermia. (**S2F Fig**). We also recovered equally high numbers of all Teff subsets when recipient mice were sacrificed 14 days after challenge (**S2G Fig**). The numbers of Teff surviving after challenge were about three logs higher than before challenge, as depicted in **S2B Fig**, suggesting that B5 TCR Tg Teff only proliferate in RAG2^o^ mice in the presence of antigen, and not homeostatically. Teff subsets had an average of 77% Teff (CD127^-^) phenotype on recovery (Teff^Early^ shown in **S2H Fig**), compared to 37% in uninfected RAG2^o^ recipients (**S2C Fig**, right plot), and were primarily Teff^Int^ and Teff^Late^. A substantial fraction of all T cells recovered after 2 weeks post-infection also produced IFN-γ (**S2I Fig**), demonstrating that all Teff subsets maintain the ability to perform effector functions even after two weeks without antigen.

**Fig 7:**
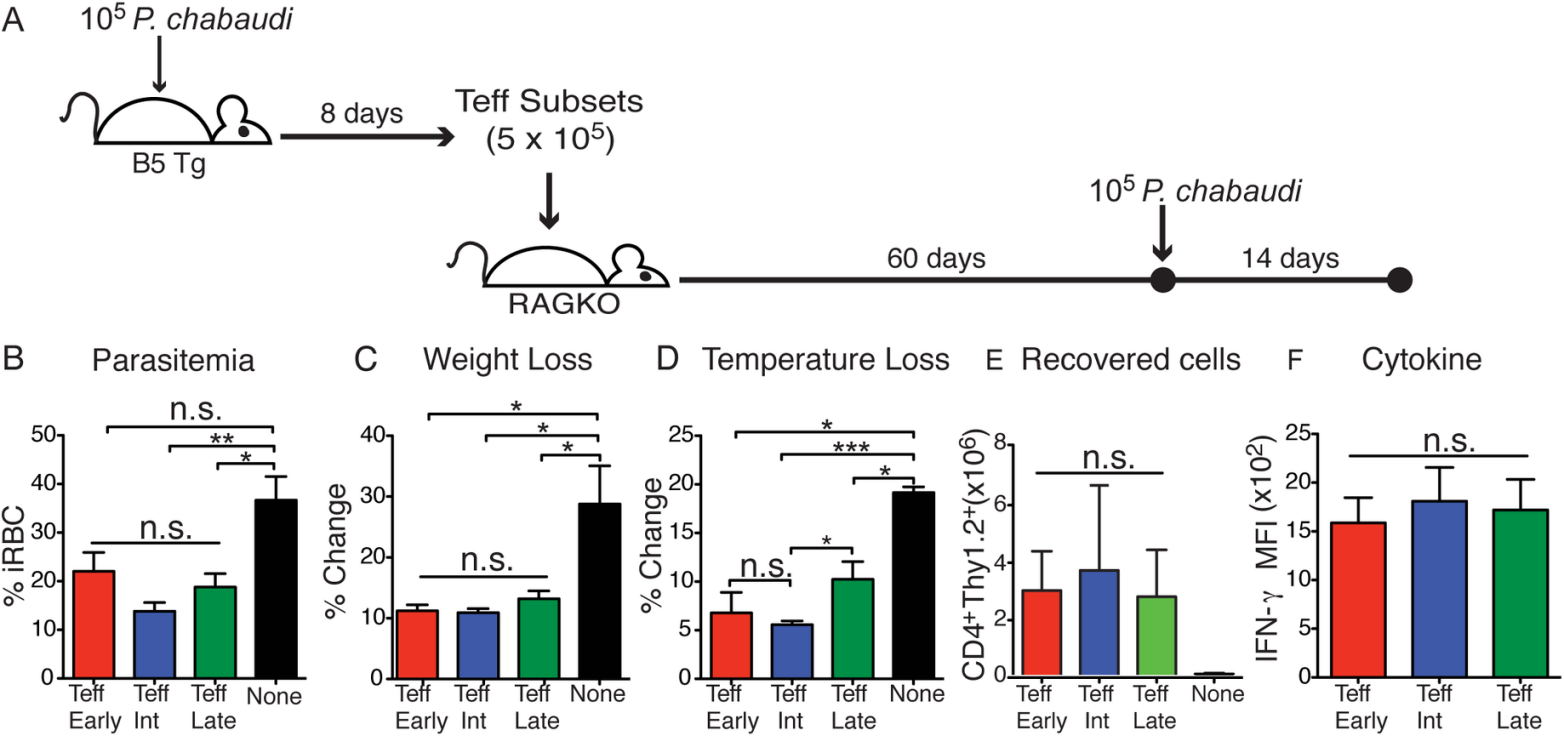
Rested effector T cells protect from parasitemia and pathology. **A)** Schematic of the experimental design. Effector T cell subsets were sorted from the spleens of B5 TCR Tg at d8 p.i. and transferred (5 x 10^5^) into RAG2^o^ mice for 60 days before infection. Recipients were then infected with 10^5^ *P*. *chabaudi*. Parasitemia, weight, and temperature change was measured for 14 days after infection. **B)** Average peak parasitemia (%iRBC/RBC) of each mouse (d8-10 p.i.) is shown. As measurements of pathology, the average of **C)** weight and **D)** temperature loss (% change) of each recipient at the peak of pathology (d8-10 p.i.) are shown. Flow cytometry was performed to quantitate **E)** the number of B5 T cells (CD4^+^Thy1.2^+^) recovered from RAG2^o^ recipients on day 14 p.i. and **F)** graph showing MFI of IFN-γ in T cells recovered from each group. Data represent 2–3 mice per group and are representative of 3 similar independent experiments. Parasitemia was analyzed using one-way ANOVA and Tukey’s post-hoc. Error bar represents SEM, * p<0.05, ** p<0.01, *** p<0.001, n.s—not significant.

In order to test the possibility that Teff can be intermediate-lived and contribute to protection even after a long absence of infection, we transferred the three Teff subsets (Teff^Early^, Teff^Int^, Teff^Late^) into RAG2^o^ recipients and infected the mice with 10^5^ parasitized RBCs after two months, as diagrammed in **Fig 7A**. Parasitemia and pathology were monitored for 14 days, and splenocytes were tested for cytokine production. Strikingly, all the three Teff subsets reduced peak parasitemia significantly **(Fig 7B)**. Interestingly, weight loss (**Fig 7C)** and hypothermia **(Fig 7D)** were significantly lower in recipients of all three Teff subsets after infection. All Teff subsets were recovered in similar large numbers after infection **(Fig 7E)**. On day 14 p.i., there was no difference in IFN-γ production observed between the groups **(Fig 7F)**. Taken together, these data suggest that dwindling Teff numbers are still able to protect from both parasitemia and pathology, plausibly by re-expanding, though we have not documented proliferation per se.

**Fig 8:**
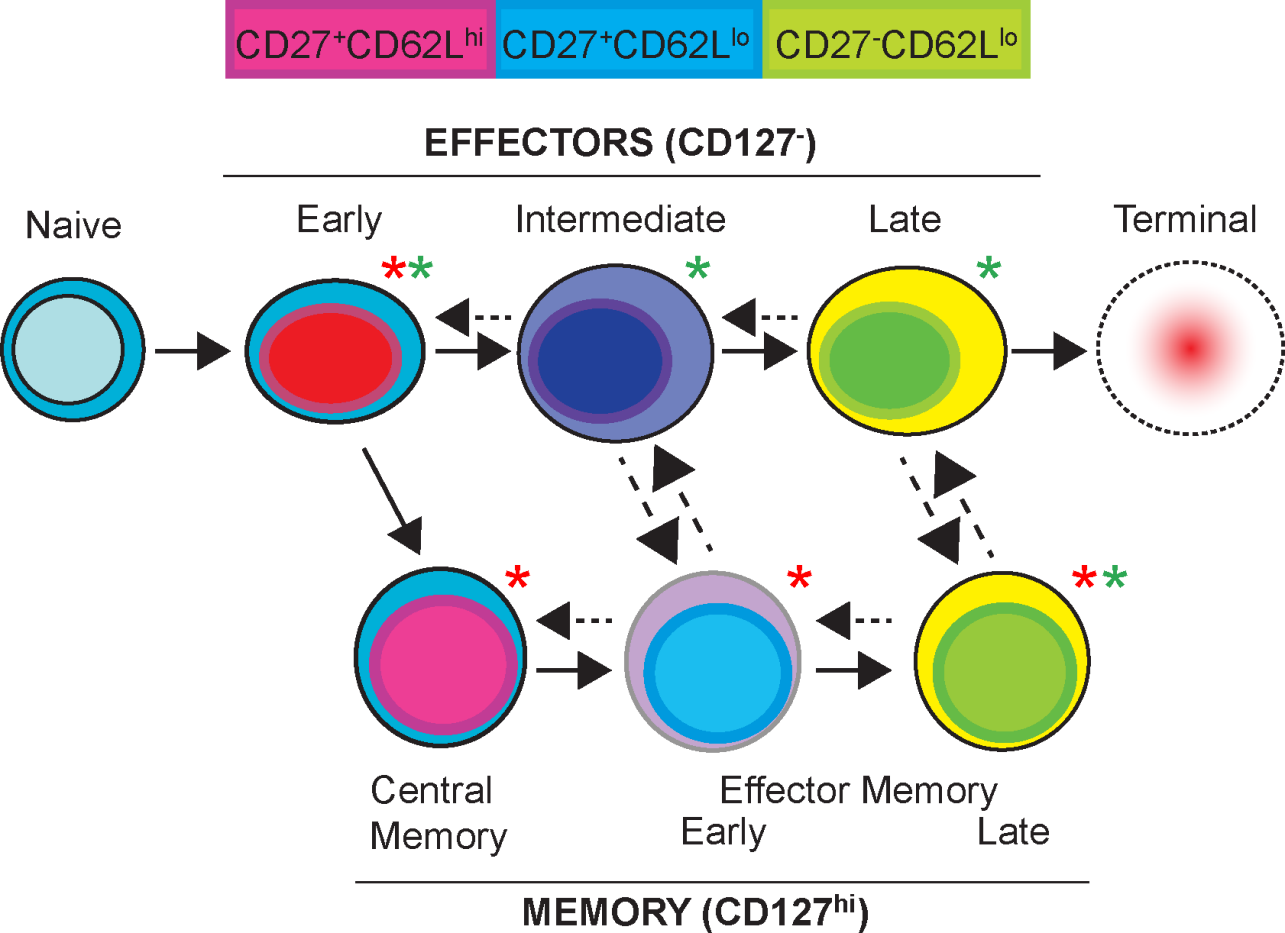
Proposed model of T cell activation and memory T cell differentiation. Upon activation of naïve T cells, Teff^Early^ (CD127^-^ CD62L^hi^ CD27^+^) are generated, which contain precursors to both effector and memory T cells. Depending on the presence of antigen (or leukopenia) in their environment, they can progress toward maturation of effector T cells, Teff^Int^, Teff^Late^ subsets, or in an uninfected environment, towards differentiation into Tcm and then Tem subsets, the latter being promoted by low-level chronicity. Upon downregulation of CD62L, Teff lose survival potential and become terminal Teff; however, surviving mature Teff can become CD127^hi^, and expand when re-activated and promote protection. Tmem can proliferate without downregulating CD127 in conditions of low antigen exposure (**Fig 4B**). Dotted arrows show less common events, or those dependent on environmental changes. Tmem can become Teff again in conditions of re-exposure to higher antigen loads (**S2B Fig**). We have also observed the more terminally differentiated subsets re-expressing CD62L, suggesting plasticity in this process. The degree of differentiation to central or effector memory T cells from Teff^Early^ is determined in the first week of *P*. *chabaudi* infection [18]. In chronic infection, and for a period after exposure to long-lasting infection, Tcm can continue to generate Tem. Populations that protect best are marked by green asterisks, and populations that survive best are marked by red asterisks.

This work has demonstrated mechanisms of Tem survival, and also contributed to our understanding of mechanisms of Tem differentiation, which we have diagrammed in **Fig 8**. We propose a model of T cell activation with the power to explain many aspects of how CD4^+^ effector memory T cells are generated in malaria infection. The model illustrates that Teff^Early^ generate Tcm, which become Tem in conditions of chronic infection. Important support for the model suggesting that Tmem differentiation occurs early after T cell activation is that while chronic *P*. *chabaudi* generates predominantly Tem cells in the long-term, the dominance of Tem over Tcm is actually determined between days 3 and 5 post-infection [12]. After this window, the ratio of these two subsets can no longer be reversed by parasite clearance to favor Tcm. Previously, we showed that Teff^Early^ could re-express CD127 and generate both Tcm and Tem in uninfected wildtype recipients, and that Tcm could make Tem subsets in the presence of high parasitemia [7]; however, it was unclear if Tcm made Tem subsets during low-level chronic infection. We confirmed that Tem^Early^ can make Tem^Late^ **(Figs 3D and 4C**) and expanded on our previous observations by showing that Tcm can be programmed by low-level chronic infection to sustain Tem cell numbers (**Fig 3D**). We also demonstrated that continuous parasite exposure after this early period further promotes Tem accumulation. Dotted arrows in the model schematic indicate plasticity in the directionality of the pathway, and these conversions are often dependent on the presence or absence of infection. For example, we previously showed transfer of Tem into infected RAG2^o^ animals with high parasitemia induced downregulation of CD127, or secondary Teff from Tem [7]; here, we showed that mature Teff do not survive well, but are nevertheless capable of re-upregulating CD127, and re-expanding to protect. In future studies, we hope to use this model to understand the mechanisms driving differentiation of protective T cell subsets.

## Discussion

A critical feature of protective T cells generated by a successful vaccination is their ability to survive long-term. To our knowledge, there is only one study in the literature that directly compares survival of CD4 central and Tem *in vivo*, and this study suffers from the difficulty of separating effector T cells from Tem resulting in the observation of a short half-life for the mixed population [21]. Using the transient but complete downregulation of CD127 by Teff has allowed us to make progress on this question with important implications for vaccine development for chronic infections. Similarly, there are no studies to our knowledge directly comparing Teff and Tem survival, though there is elegant work comparing effector function and protection of activated subsets in various models [13]. The current paradigm suggests that Tmem, even CD62L^lo^ Tem, might survive more durably than CD127^**-**^ Teff in the absence of persistent infection, and indeed our data support this hypothesis. Interestingly, CD27^-^ CD8^+^ T cells have been previously reported to be protective, intermediate-lived, and proliferate to self-renew [17, 27]. However, our data show that the protective CD4^+^ CD127^hi^ CD27^-^ Tem^Late^ definitely survives longer than CD127^**-**^ CD27^**-**^ Teff. This suggests that the shorter half-life reported could be due to the inclusion of both CD127^-^ CD27^-^ Teff and CD127^+^ CD27^-^ T cells. Nevertheless, our data are compatible with the interpretation that in the long-term, both Teff^Late^ and Tem^Late^ decay faster than other Teff and Tmem T cell subsets, respectively.

Tcm are known to survive through homeostatic proliferation [15]. However, Tem are not perpetuated by this slow, cytokine-driven turnover mechanism. On the other hand, T resident memory cells (Trm), which have a similar surface phenotype to Tem, but do not recirculate, clearly do survive in peripheral tissues for prolonged periods in the absence of antigen [27, 28]. The current paradigm for Tem survival is that Tem predominate in chronic infections in an antigen-dependent manner, suggesting a requirement for antigen [7, 29]. However, we show here that Tem were able to survive in uninfected recipients similar to Tcm, suggesting a variety of unknown survival mechanisms. Our previous study showed that Tem are preferentially generated (over Tcm) during *P*. *chabaudi* infection lasting longer than three days, showing that Tem generation is not only a result of long-term infection [12]. In the current studies, we have added three additional later mechanisms that promote a high Tem to Tcm ratio after the initial activation event in addition to unexpected Tem survival. We have also shown that CD4 Tcm can generate Tem in the presence of high-level chronic infection in immunodeficient animals [7]. Here, we observed that Tcm purified from persistently infected donors can also differentiate into Tem in uninfected recipients (**Fig 3D**). This transition of Tcm to Tem subsets occurs even when the donors are treated to clear the infection. Therefore, progressive differentiation of memory T cells from Tcm to Tem^Late^ can be pre-programmed during chronic infection and continue even after clearance. It is formally possible that the CD62L^lo^ and CD27^-^ cells observed in these experiments are the result of surface cleavage of these two molecules, which are regulated by proteolytic cleavage; however, samples were always handled on ice as a technical precaution to avoid this artifact. In addition, similar forward differentiation of Tcm into Tem has been reported in studies of CD8 T cells [30]. Some studies have also proposed the reverse direction of differentiation of CD8 T cells back from Tem into Tcm, which we also see in some experiments, though to a lesser degree, and this reversion is not the result of enzymatic shedding of CD62L [31–33]. In our work, this conclusion is further strengthened by the observation that reducing the length of time of exposure of Tcm cells to persistent infection, by treatment of donors with an anti-malarial drug, significantly reduces progression of Tem^Early^ to Tem^Late^. There is significant regulation of lifespan and proliferation in the downregulation of CD27 in CD8 T cells, particularly memory [34].

The second additional mechanism of Tem predominance that we observed is that persistent infection increased the overall survival of Tem^Early^ by inducing proliferation and expansion, without differentiation of these cells into CD127- Teff (**Fig 4B and 4C**). This may help to explain the poor protection of the natural memory T cell population. Interestingly, Tem^Early^ represent about half of the memory T cells late in infection, while Tem^Late^ are fewer [7]. The higher representation of Tem^Early^ supports the trend apparent in our data that Tem^Late^ do not persist as well as Tem^Early^, even during persistent infection. Therefore, while we show that chronic infection is not required for the survival of Tem, we have also uncovered three potential mechanisms explaining how pathogen persistence promotes an increased representation of CD62L^lo^ CD127^hi^ (CD27-) CD44^hi^ Tem in the Tmem pool.

The third mechanism of Tem maintenance in chronic infection suggested by our data, is the potential for mature Teff to contribute to the generation of Tem, which is suggested by the CD127^hi^ phenotypes seen on recovery of all Teff subsets from uninfected Thy1.1 hosts after 60 days (**Fig 2C**). We also previously showed an intermediate level of CD127 re-upregulation on Teff^Early^ recovered from uninfected congenic hosts after two weeks [12]. This observation supports the dominant paradigm that Tem are also generated from Teff that survive after contraction [35], though highly differentiated Teff lose their Tmem potential [12, 36]. The caveat to the interpretation that CD127 (IL-7Rα) re-upregulation indicates Tmem differentiation, is that CD127 downregulation is known to be transient. However, re-upregulation of CD127 in CD8 Teff was recently shown to correspond with an epigenetic program of de-differentiation at some loci of Teff into a more resting state [33]. Furthermore, we showed here that Teff still have the potential to protect recipients after two months, a feature of memory T cells. However, it is important to note that survival of Teff is much lower than Tem, which along with the early timepoint of the programming of Tem suggests an important contribution to Tem generation from CD62L^hi^ precursors.

The traditional target for vaccination has been to generate long-lived Tmem. However, the protection provided to the host by a single infection is not complete. This is particularly obvious in people living in malaria endemic areas who are exposed to sequential heterologous *Plasmodium* infections, but remain susceptible to malaria. Poor immunity suggests that either generation of memory B or T cells, or the quality of the adaptive immune response is sub-optimal. We have studied the functionality of the fairly protective mixed effector/memory T cell population present two months after chronic *P*. *chabaudi* infection [37], and showed that they do not re-expand on homologous re-infection, but that they can make cytokines. In order to understand the potential of Teff and Tmem cells to contribute to protection, we tested the protective potential of CD127^hi^ Tmem subsets, including Tem. Upon *P*. *chabaudi* infection of recipients of the Tmem subsets, we observed a small reduction in parasitemia only in animals that received the Tem^Late^ (CD127^hi^CD62L^lo^CD27^-^) subset, but not Tcm or Tem^Early^. Both Tcm and Tem^Late^ reduced hypothermia in infected recipients. Other studies have shown that CD8^+^ CD27^-^, similar to the Tem^Late^ phenotype, can protect mice from *Listeria* infection, though it is not clear if these are primarily Teff or Tmem [38]. Significantly, Tem^Early^, the cell type making up the largest fraction of Tmem after *P*. *chabaudi* infection [7], had no beneficial effect on either parasitemia or pathology, but still promoted survival of the hosts. This could contribute to the poor protection most notable on re-infection with heterologous parasite. Therefore, our data suggest that while specific Tmem may not be very efficient at controlling acute infection, the right types of Tem can reduce clinical manifestations of disease and allow the host to survive. Though we have previously shown that each Tmem subset has a unique cytokine profile [7], it is not yet clear what effector functions of Tem^Late^ contribute to their unique protective phenotype. In wildtype mice and humans, levels of pre-existing antibodies are very likely to contribute to the effector functions promoted by memory CD4 T cells, suggesting important interactions. However, we do not have a model system at this time to evaluate the contribution of T cells in the context of pre-existing serum antibody or pre-activated innate cells.

Strikingly, all CD127^-^ effector T cell subsets contribute to a striking reduction in peak parasitemia in infected hosts. The Teff^Late^ population produced higher proportions of all three cytokines. However, the number of triple-cytokine producers (TNF^+^IFN-γ^+^IL-2^+^) was similar in all recipient groups, suggesting a mechanism for the equal protection provided, as multi-cytokine producers correlate with protection in several infections [26]. Overall, we conclude from our studies that effector T cells provide better protection compared to the universal standard, than memory T cells. Several other studies have shown important protective effects of Teff, especially in chronic infection [13, 38, 39]. An important conclusion from earlier studies is that chronic infection promotes maintenance of Teff cell numbers. Teff protect well, but have a shorter half-life, potentially explaining the decay of T cell immunity seen over time as parasitemia decays in this infection and other persistent infections [6]; however, this hypothesis remains to be tested *in vivo*. In that context, it is notable that not all Teff subsets decay at the same rate. The Teff^Early^ subset is particularly interesting, as this subset protects like other Teff, but Teff^Early^ also survive in higher numbers than terminally differentiated Teff^Late^ in replete hosts without infection over two months. We previously showed that Teff^Early^ can generate all other Teff and Tmem subsets [12]. In contrast, the more mature or activated Teff^Int^ and Teff^Late^ progressively lose the potential to generate memory T cells in both RAG2^o^ and T cell replete hosts. Progressive loss of memory potential with increasing time of exposure to inflammation has been hypothesized for CD8 effector T cells [40].

In conclusion, the current study defines two CD4 T cell populations (Teff^Early^ and Tem^Late^) that are both long-lived and protect against malaria infection, though to different degrees. These phenotypes could represent correlates of immunity, or targets for vaccination. The observation that short-lived Teff present at the peak of malaria infection protect better than long-lived Tmem that predominate at later timepoints suggests a decay of effector function that could explain the decline in clinical immunity especially in the absence of exposure. Therefore, a better understanding of the mechanisms for the survival of these subsets, and their effector functions *in situ*, is critical. Better understanding of the functions and generation of the diverse T cell populations in the memory phase of this infection could help us design more effective vaccines that generate long-lived, protective T cells in chronic infection.

## Materials and methods

### Ethics statement

All animal experiments were carried out according to protocol number 1006031A, as reviewed and approved by the University of Texas Medical Branch Institutional Animal Care and Use Committee (IACUC). The studies were performed in accordance with the guidelines in the Guide for the Care and Use of Laboratory Animals, 8th edition (Institute of Laboratory Animal Resources, National Academies Press, Washington, DC) and regulatory document from Public Health Service (PHS) Policy on the Humane care and use of Laboratory Animals.

### Mice and parasites

Thy1.1 BALB/cByJ were backcrossed to BALB/cJ (N4; The Jackson Laboratory, Bar Harbor, ME). B5 TCR Tg mice, a kind gift from J. Langhorne (Francis Crick Institute, London, UK), were generated as previously described [25] and backcrossed to BALB/cJ (N7-10) and maintained in the UTMB Animal Resources Center. The B5 TCR recognizes MSP-1 (1157–1171, ISVLKSRLLKRKKYI/I-E^d^); B5 TCR Tg mice were typed using primers Vα2, 5’- gaacgttccagattccatgg-3’ and 5’-atggacaagatcctgacagcatcg-3’, and Vβ8.1, 5’-cagagaccctcaggcggctgctcagg-3’ and 5’- atgggctccaggctgttctttgtggttttgattc-3’. RAG2^0^ (Taconic, Germantown, NY) were used at 9–12 weeks old. All other mice were used at 6–12 weeks old and infected with 10^5^ (expect in RAG2^0^ infection 5x10^4^) *Plasmodium chabaudi chabaudi* (AS)-infected erythrocytes i.p. (kind gift of J. Langhorne). Parasites were counted by light microscopy in thin blood smears stained with Giemsa (Sigma-Aldrich, St. Louis, MO) [41]. In some experiments, mice were treated with a dose of 50 mg/kg of the antimalarial drug chloroquine (CQ, i.p.) on days 30–34 post-infection (p.i.). C57BL/6 mice were purchased from Jackson Labs (Bar Harbor, ME) and were treated with a dose of 20 mg/kg off the antimalarial drug mefloquine chloride (MQ, i.t., from 4 mg/ml stock) on days 10–14 for the effector time-course study and days 30–34 for the memory time-course study. All adoptive transfer experiments were done using age-matched donors.

### Flow cytometry

Single-cell suspensions of splenocytes were made in HEPES-buffered HBSS (Mediatech, Manassas, VA), then depleted of erythrocytes by incubation in RBC lysis buffer (eBioscience, San Diego, CA). For all Figures except **Fig 1**, Thy1.2^+^ T cells were enriched by positive selection using Miltenyi Thy1.2 microbeads (San Diego, CA), double stained and analyzed using the “dump” gate to get maximal resolution. Cells were then stained in PBS with 2% FBS (Sigma, St. Louis, MO) and 0.1% sodium azide with anti-CD16/32 (2.4G2) supernatant (BioXCell, West Lebanon, NH) for Fc receptor blocking, followed by double staining for Thy1.2 in both -FITC, -PE, and a combination of other PerCP-Cy5.5, PE/cyanine 7 (Cy7), PE/Cy5, Allophycocyanin (APC), or APC/efluor780–conjugated Abs (all from eBioscience); or CD62L PE-Texas Red (Invitrogen, Life Technologies). A combination of CD11b, F4/80, and Ter119 biotin antibodies followed by streptavidin-PerCyP-Cy5.5, were used as a “dump” channel for analysis of donor cells in RAG2^o^ mice. Cells were collected on a LSRII Fortessa using FACSDiva software (BD Biosciences, San Jose, CA) and analyzed in FlowJo (version 9.7, Tree Star, Ashland, OR). Compensation was performed in FlowJo using single-stained splenocytes (using CD4 in all colors). Data from each mouse was analyzed and averages and SEM calculated in Excel (Microsoft). Data from three to four mice are concatenated in some figures to achieve sufficient cell numbers for presentation.

For intracellular cytokine staining, 5 x 10^6^ cells/ml were stimulated with PMA and ionomycin at 37°C for 5 hours and Brefeldin A was added (all from Sigma) for the last two hours. Cells were harvested and processed for surface staining as described above. Cells were fixed with 2% paraformaldehyde and permeabilized using BD perm buffer (BDbiosciences). Cells were washed 3 times and stained for IFN-γ, IL-2 or TNF or isotype controls. Cell Trace Violet (CFSE, Invitrogen, Carlsbad, CA) was used according to manufacturers’ instructions. Sorted cells were washed twice with PBS without calcium and magnesium and incubated with 1x dilution of cell trace violet at 37°C in a water bath for 10 minutes while shaking. Labeled cells were transferred into recipient mice at 5x10^4^–2.5x10^5^ cells (see Results and Legends for number of cells in each experiment). Except for **Fig 5C**, which is calculated as the % recovered Tg cells/lymphocytes x the number of lymphocytes, recovered cells were counted by inclusion of counting beads (AccuCheck, Molecular Probes, ThermoFisher Scientific) in the FACS sample according to manufacturer’s instructions and calculated using the equation: Total cell number = ((all cell event count / bead event count) x Bead conc. (per ml) x (Bead volume / cell volume)) x Volume of original sample. This formula only works if the whole volume of FACS tube is collected.

### Cell sorting and protection assay

Single cell suspensions of splenic CD4^+^ T cells from (10–12) infected (d8 for Teff; d60 for Tmem) B5 TCR Tg donors were enriched using negative selection with EasySep biotin microbeads (Stemcell Technologies, Vancouver, BC, Canada) with biotinylated anti-CD8a (55–6.7), B220 (RA3-6B2), CD11b (MI/70), CD11c (N418), F4/80 (BM8), and Ter119 (eBioscience, San Diego, CA). Enriched T cells were then stained with anti-CD4-FITC, CD44-APC/Cy7, CD127-PE, CD62L-Texas Red and CD27-APC for Teff or Tmem subset sorts. Cells were sorted on a FACSAria with FACSDiva software (BDbiosciences) to >99% purity (as shown in **S1 Fig**). B cells were purified from BALB/cJ mice that were infected with *P*. *chabaudi* twice over a two-month period using CD19 microbeads from Miltenyi (San Diego, CA) (98% pure). For protection assays, RAG2^o^ mice (Jackson Laboratory) were given sort-purified T cells (2.5x10^5^) together with immune B cells (1–2 x 10^7^, i.p.), and were infected with 5x10^4^ *P*. *chabaudi* infected RBCs. Weight and body temperature were measured every other day using a portable Ohaus balance (Parsippany, NJ), and temperature microchip transponders (IPTT-300, BMDS, Seaford, DE)

### Statistics

Where indicated, experiments were analyzed by one-way ANOVA, followed by Tukey’s for nonparametric data or the Student’s *t* test for parametric data in Prism (GraphPad, La Jolla, CA): *p ≤ 0.05, **p ≤ 0.01, ***p ≤ 0.001. Limit of detection is defined as three times the standard deviation of the blank. Therefore, the limit of detection for the transferred Thy1.1+ cells in all experiments was calculated using all available data from animals from all experiments that did not receive transferred T cells.

## Supporting information

S1 Fig Gating and purity of effector and memory T cell subsets. Effector or memory T cells were sorted from 10–12 pooled spleens from infected (**A**) day 60, Tmem, or (**B**) d8, Teff *P*. *chabaudi*-infected B5 TCR Tg mice. (**A, B)** In all data shown, and in the cell sorting, CD4^+^ T cells were gated for singlet discrimination using side and forward scatter characteristics. CD4^+^ cells were identified on a histogram after magnetic bead purification. Contour plots and histograms represent pre (top) and post- (bottom) sort of **A)** memory (CD44^hi^CD127^hi^) or **B**) effector (CD44^hi^CD127^-^) T cell phenotypes used to isolate individual subsets by CD62L and CD27.
(TIF)

S2 Fig “Rested” Teff^Early^ cells re-upregulate CD127 in RAG2^o^ mice and differentiate. **A)** Schematic of experimental design. (**B-I**) Effector T cell subsets were sorted from the spleens of B5 TCR Tg at d8 p.i. and transferred (5x10^5^) into RAG2^o^ mice for 14 days. (**E-I**) Fourteen days post-transfer, the RAG2^o^ recipients were infected with *P*. *chabaudi*. **B)** Graph showing numbers of recovered B5 T cells (CD4^+^ Thy1.2^+^) per spleen in each group of recipients at day 14 post-transfer. (**C, D**) Contour plots and summary graphs showing transferred (IN) and recovered (OUT) Teff^Early^ (CD127^-^ CD62L^hi^ CD27^+^) before infection. **C)** Proportions of Teff (CD127^-^) or Tmem (CD127^hi^) populations and **D)** Teff and Tmem subset phenotype (CD62L, CD27) of recovered B5 T cells on day 14 post-transfer are shown. (**E-I**) Infected recipient mice are shown at the peak of each symptom for each recipient (d8-10 p.i.). Graphs of **E)** Parasitemia (%iRBC/RBC), and **F)** percent weight loss and hypothermia are shown. **G)** Graph of average number of recovered B5 Tg T cells. **H)** Contour plot showing phenotype of Teff and subsets from Teff^Early^ OUT B5 T cells recovered from infected RAG mice, and **I)** Average Percent IFN-γ+ of B5 TCR Tg T cells recovered day 14 p.i. are shown. Contour plots are representative of 3 mice per group from 3 independent experiments. Data was analyzed in Prism using One-way ANOVA followed by Tukey’s and Students t-test for cell numbers. Error bars represent SEM, * p<0.05, ** p<0.01, *** p<0.001, and n.s.–not significant.
(TIF)

## References

[1] WHO. World Malaria Report 2015. 280 p.

[2] Doolan DL, Dobaño C, Baird JK. Acquired Immunity to Malaria. Clinical Microbiology Reviews. 2009;22(1):13–36. doi: 10.1128/CMR.00025-08 19136431 10.1128/CMR.00025-08 PMC2620631

[3] Collins WE, Jeffery GM. A retrospective examination of the patterns of recrudescence in patients infected with *Plasmodium falciparum*. Am J Trop Med Hyg. 1999;61:44–8 . 10432044 10.4269/tropmed.1999.61-044

[4] Hamad AA, El Hassan IM, El Khalifa AA, Ahmed GI, Abdelrahim SA, Theander TG, et al Chronic *Plasmodium falciparum* infections in an area of low intensity malaria transmission in the Sudan. Parasitology. 2000;120 Pt 5):447–56 . 10840974 10.1017/s0031182099005818

[5] Achtman AH, Stephens R, Cadman ET, Harrison V, Langhorne J. Malaria-specific antibody responses and parasite persistence after infection of mice with *Plasmodium chabaudi chabaudi*. Parasite Immunol. 2007;29(9):435–44. doi: 10.1111/j.1365-3024.2007.00960.x . 17727567 10.1111/j.1365-3024.2007.00960.x

[6] Freitas do Rosario AP, Muxel SM, Rodriguez-Malaga SM, Sardinha LR, Zago CA, Castillo-Mendez SI, et al Gradual decline in malaria-specific memory T cell responses leads to failure to maintain long-term protective immunity to *Plasmodium chabaudi AS* despite persistence of B cell memory and circulating antibody. J Immunol. 2008;181(12):8344–55. doi: 10.1371/journal.ppat.1001208 19050251 10.4049/jimmunol.181.12.8344

[7] Stephens R, Langhorne J. Effector memory Th1 CD4 T cells are maintained in a mouse model of chronic malaria. PLoS Pathogens. 2010;6(11):e1001208 doi: 10.1371/journal.ppat.1001208 . 21124875 10.1371/journal.ppat.1001208 PMC2991260

[8] White MT, Verity R, Griffin JT, Asante KP, Owusu-Agyei S, Greenwood B, et al Immunogenicity of the RTS,S/AS01 malaria vaccine and implications for duration of vaccine efficacy: secondary analysis of data from a phase 3 randomised controlled trial. The Lancet Infectious Diseases. 2015;15(12):1450–8. doi: 10.1016/S1473-3099(15)00239-X 26342424 10.1016/S1473-3099(15)00239-X PMC4655306

[9] Olotu A, Fegan G, Wambua J, Nyangweso G, Awuondo KO, Leach A, et al Four-Year Efficacy of RTS,S/AS01E and Its Interaction with Malaria Exposure. N Eng J Med. 2013;368(12):1111–20. doi: 10.1056/NEJMoa1207564 . 23514288 10.1056/NEJMoa1207564 PMC5156295

[10] Seder RA, Chang LJ, Enama ME, Zephir KL, Sarwar UN, Gordon IJ, et al Protection against malaria by intravenous immunization with a nonreplicating sporozoite vaccine. Science. 2013;341(6152):1359–65. doi: 10.1126/science.1241800 . 23929949 10.1126/science.1241800

[11] Mordmuller B, Surat G, Lagler H, Chakravarty S, Ishizuka AS, Lalremruata A, et al Sterile protection against human malaria by chemoattenuated PfSPZ vaccine. Nature. 2017;542(7642):445–9. doi: 10.1038/nature21060 . 28199305 10.1038/nature21060 PMC10906480

[12] Opata MM, Carpio VH, Ibitokou SA, Dillon BE, Obiero JM, Stephens R. Early Effector Cells Survive the Contraction Phase in Malaria Infection and Generate Both Central and Effector Memory T Cells. J Immunol. 2015;194(11):5346–54. doi: 10.4049/jimmunol.1403216 . 25911759 10.4049/jimmunol.1403216 PMC4433766

[13] Peters NC, Pagan AJ, Lawyer PG, Hand TW, Henrique Roma E, Stamper LW, et al Chronic parasitic infection maintains high frequencies of short-lived Ly6C+CD4+ Effector T cells that are required for protection against re-infection. PLoS Pathog. 2014;10(12):e1004538 doi: 10.1371/journal.ppat.1004538 . 25473946 10.1371/journal.ppat.1004538 PMC4256462

[14] Carpio VH, Opata MM, Montanez ME, Banerjee PP, Dent AL, Stephens R. IFN-gamma and IL-21 Double Producing T Cells Are Bcl6-Independent and Survive into the Memory Phase in *Plasmodium chabaudi* Infection. PLoS One. 2015;10(12):e0144654 doi: 10.1371/journal.pone.0144654 . 26646149 10.1371/journal.pone.0144654 PMC4672895

[15] Geginat J, Lanzavecchia A, Sallusto F. Proliferation and differentiation potential of human CD8+ memory T-cell subsets in response to antigen or homeostatic cytokines. Blood. 2003;101(11):4260–6. doi: 10.1182/blood-2002-11-3577 . 12576317 10.1182/blood-2002-11-3577

[16] Roberts A, Ely K, Woodland D. Differential contributions of central and effector memory T cells to recall responses. J Exp Med. 2005;202(1):123–33. doi: 10.1084/jem.20050137 . 15983064 10.1084/jem.20050137 PMC2212898

[17] Nolz JC, Rai D, Badovinac VP, Harty JT. Division-linked generation of death-intermediates regulates the numerical stability of memory CD8 T cells. Proc Natl Acad Sci 2012;109(16):6199–204. doi: 10.1073/pnas.1118868109 . 22474367 10.1073/pnas.1118868109 PMC3341021

[18] Opata MM, Stephens R. Early Decision: Effector and Effector Memory T Cell Differentiation in Chronic Infection. Curr Immunol Rev. 2013;9(3):190–206. doi: 10.2174/1573395509666131126231209 . 24790593 10.2174/1573395509666131126231209 PMC4000274

[19] Kaech SM, Wherry EJ, Ahmed R. Effector and memory T-cell differentiation: implications for vaccine development. Nat Rev Immunol. 2002;2(4):251–62. doi: 10.1038/nri778 . 12001996 10.1038/nri778

[20] McDermott DS, Varga SM. Quantifying antigen-specific CD4 T cells during a viral infection: CD4 T cell responses are larger than we think. J Immunol. 2011;187(11):5568–76. doi: 10.4049/jimmunol.1102104 . 22043009 10.4049/jimmunol.1102104 PMC3221938

[21] Macallan DC, Wallace D, Zhang Y, De Lara C, Worth AT, Ghattas H, et al Rapid turnover of effector-memory CD4(+) T cells in healthy humans. J Exp Med. 2004;200(2):255–60. doi: 10.1084/jem.20040341 . 15249595 10.1084/jem.20040341 PMC2212011

[22] Hunt P, Cravo PV, Donleavy P, Carlton JM, Walliker D. Chloroquine resistance in *Plasmodium chabaudi*: are chloroquine-resistance transporter (crt) and multi-drug resistance (mdr1) orthologues involved? Mol Biochem Parasitol. 2004;133(1):27–35. doi: 10.1038/nm0402-379 14668009 10.1016/j.molbiopara.2003.08.010

[23] Martin DL, Tarleton RL. Antigen-specific T cells maintain an effector memory phenotype during persistent *Trypanosoma cruzi* infection. J Immunol. 2005;174(3): 1594–601. . 15661921 10.4049/jimmunol.174.3.1594

[24] Appay V, Dunbar P, Callan M, Klenerman P, Gillespie G, Papagno L, et al Memory CD8+ T cells vary in differentiation phenotype in different persistent virus infections. Nat Med. 2002;8(4):379–85. doi: 10.1038/nm0402-379 . 11927944 10.1038/nm0402-379

[25] Stephens R, Albano F, Quin S, Pascal B, Harrison V, Stockinger B, et al Malaria-specific transgenic CD4+ T cells protect immunodeficient mice from lethal infection and demonstrate requirement for a protective threshold of antibody production for parasite clearance. Blood. 2005;106(5):1676–84. doi: 10.1182/blood-2004-10-4047 . 15890689 10.1182/blood-2004-10-4047

[26] Seder R, Darrah P, Roederer M. T-cell quality in memory and protection: implications for vaccine design. Nat Rev Immunol. 2008;8(4):247–58. doi: 10.1038/nri2274 . 18323851 10.1038/nri2274

[27] Hikono H, Kohlmeier J, Takamura S, Wittmer S, Roberts A, Woodland D. Activation phenotype, rather than central- or effector-memory phenotype, predicts the recall efficacy of memory CD8+ T cells. J Exp Med. 2007;204(7):1625–36. doi: 10.1084/jem.20070322 . 17606632 10.1084/jem.20070322 PMC2118640

[28] Gebhardt T, Mackay LK. Local immunity by tissue-resident CD8+ memory T cells. Front Immunol. 2012;3:340 doi: 10.3389/fimmu.2012.00340 . 23162555 10.3389/fimmu.2012.00340 PMC3493987

[29] Martin DL, Tarleton RL. Antigen-Specific T Cells Maintain an Effector Memory Phenotype during Persistent *Trypanosoma cruzi* Infection. The Journal of Immunology. 2005;174(3):1594–601. doi: 10.1038/ni889 15661921 10.4049/jimmunol.174.3.1594

[30] Buchholz VR, Gräf P, Busch DH. The smallest unit: effector and memory CD8(+) T cell differentiation on the single cell level. Front Immunol. 2013;4 doi: 10.3389/fimmu.2013.00031 . 23424063 10.3389/fimmu.2013.00031 PMC3573211

[31] Wherry E, Teichgraber V, Becker T, Masopust D, Kaech S, Antia R, et al Lineage relationship and protective immunity of memory CD8 T cell subsets. Nat Immunol. 2003;4(3):225–34. doi: 10.1038/ni889 . 12563257 10.1038/ni889

[32] Zaph C, Rook K, Goldschmidt M, Mohrs M, Scott P, Artis D. Persistence and Function of Central and Effector Memory CD4+ T Cells following Infection with a Gastrointestinal Helminth. J Immunol. 2006;177(1):511–8. doi: 10.1038/80877 16785548 10.4049/jimmunol.177.1.511 PMC1805702

[33] Youngblood B, Hale JS, Kissick HT, Ahn E, Xu X, Wieland A, et al Effector CD8 T cells dedifferentiate into long-lived memory cells. Nature. 2017 doi: 10.1038/nature25144 10.1038/nature25144 PMC5965677 29236683

[34] Hendriks J, Gravestein L, Tesselaar K, van Lier R, Schumacher T, Borst J. CD27 is required for generation and long-term maintenance of T cell immunity. Nat Immunol. 2000;1(5):433–40. doi: 10.1038/80877 . 11062504 10.1038/80877

[35] Kurtulus S, Tripathi P, Hildeman DA. Protecting and rescuing the effectors: roles of differentiation and survival in the control of memory T cell development. Front Immunol. 2012;3 doi: 10.3389/fimmu.2012.00404 . 23346085 10.3389/fimmu.2012.00404 PMC3552183

[36] Joshi N, Cui W, Chandele A, Lee H, Urso D, Hagman J, et al Inflammation Directs Memory Precursor and Short-Lived Effector CD8+ T Cell Fates via the Graded Expression of T-bet Transcription Factor. Immunity. 2007;27(2):281–95. doi: 10.1016/j.immuni.2007.07.010 17723218 10.1016/j.immuni.2007.07.010 PMC2034442

[37] Opata MM, Stephens R. Chronic *Plasmodium chabaudi* Infection Generates CD4 Memory T Cells with Increased T Cell Receptor Sensitivity but Poor Secondary Expansion and Increased Apoptosis. Infection and Immunity. 2017;85(3). doi: 10.1016/j.immuni.2013.05.009 10.1128/IAI.00744-16 PMC5328498 28031266

[38] Olson JA, McDonald-Hyman C, Jameson SC, Hamilton SE. Effector-like CD8(+) T cells in the memory population mediate potent protective immunity. Immunity. 2013;38(6):1250–60. doi: 10.1016/j.immuni.2013.05.009 . 23746652 10.1016/j.immuni.2013.05.009 PMC3703254

[39] Scott P, Artis D, Uzonna J, Zaph C. The development of effector and memory T cells in cutaneous leishmaniasis: the implications for vaccine development. Immunol Rev. 2004;201:318–38. doi: 10.1111/j.0105-2896.2004.00198.x . 15361250 10.1111/j.0105-2896.2004.00198.x

[40] Intlekofer AM, Takemoto N, Wherry EJ, Longworth SA, Northrup JT, Palanivel VR, et al Effector and memory CD8+ T cell fate coupled by T-bet and eomesodermin. Nat Immunol. 2005;6(12):1236–44. doi: 10.1038/ni1268 . 16273099 10.1038/ni1268

[41] Li C, Corraliza I, Langhorne J. A defect in interleukin-10 leads to enhanced malarial disease in *Plasmodium chabaudi chabaudi* infection in mice. Infect Immun. 1999;67(9):4435–42. . 10456884 10.1128/iai.67.9.4435-4442.1999 PMC96762

