# Supplementary figures and images for: Protection by and maintenance of CD4 effector memory and effector T cell subsets in persistent malaria infection
Source: PLoS Pathog. 2018 Apr 9;14(4):e1006960. doi: 10.1371/journal.ppat.1006960 (PMC5908200; doi:10.1371/journal.ppat.1006960)

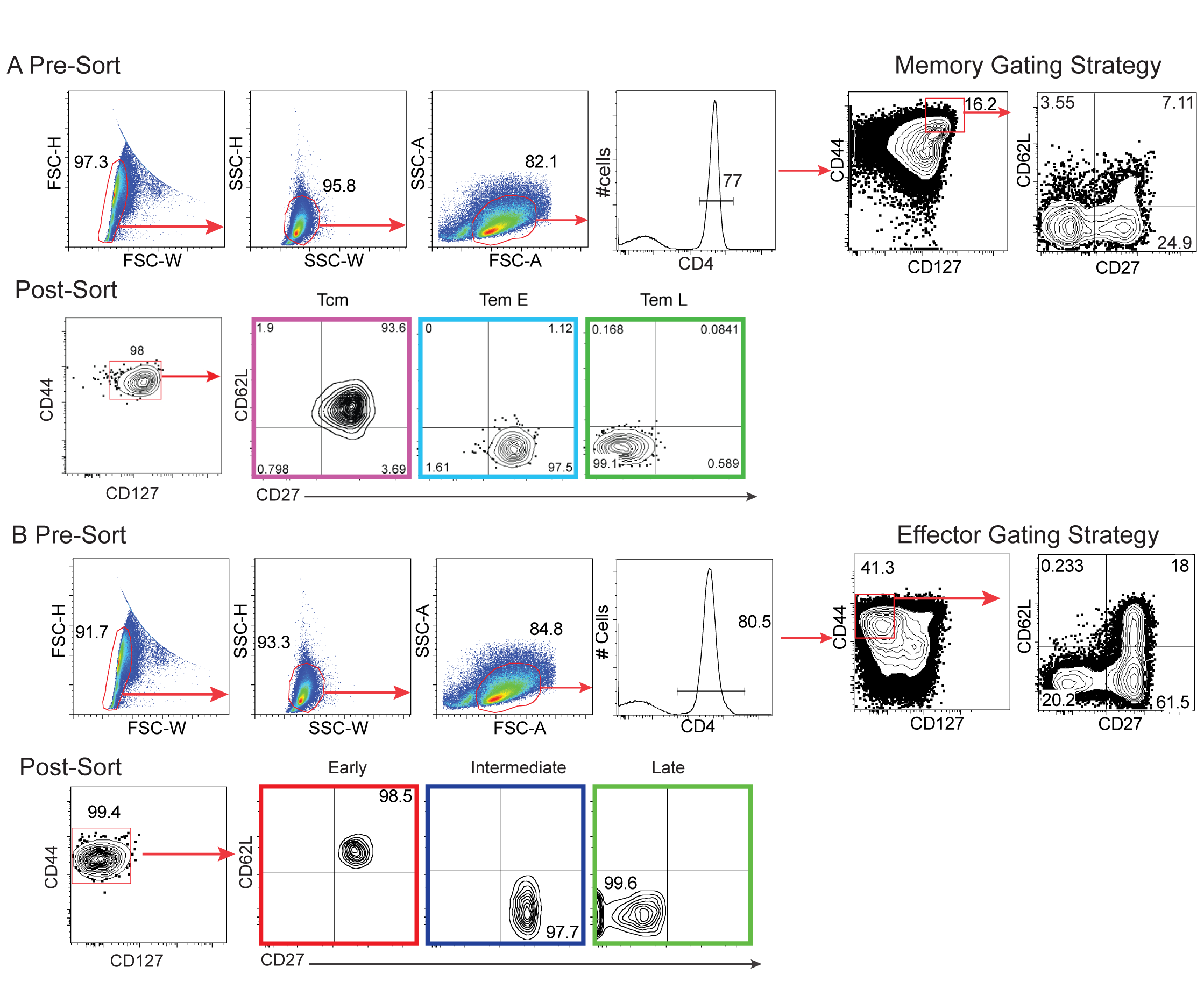

Supplement: S1 Fig — Effector or memory T cells were sorted from 10–12 pooled spleens from infected (A) day 60, Tmem, or (B) d8, Teff P. chabaudi-infected B5 TCR Tg mice. (A, B) In all data shown, and in the cell sorting, CD4+ T cells were gated for singlet discrimination using side and forward scatter characteristics. CD4+ cells were identified on a histogram after magnetic bead purification. Contour plots and histograms represent pre (top) and post- (bottom) sort of A) memory (CD44hiCD127hi) or B) effector (CD44hiCD127-) T cell phenotypes used to isolate individual subsets by CD62L and CD27. (TIF) [file ppat.1006960.s001.tif]

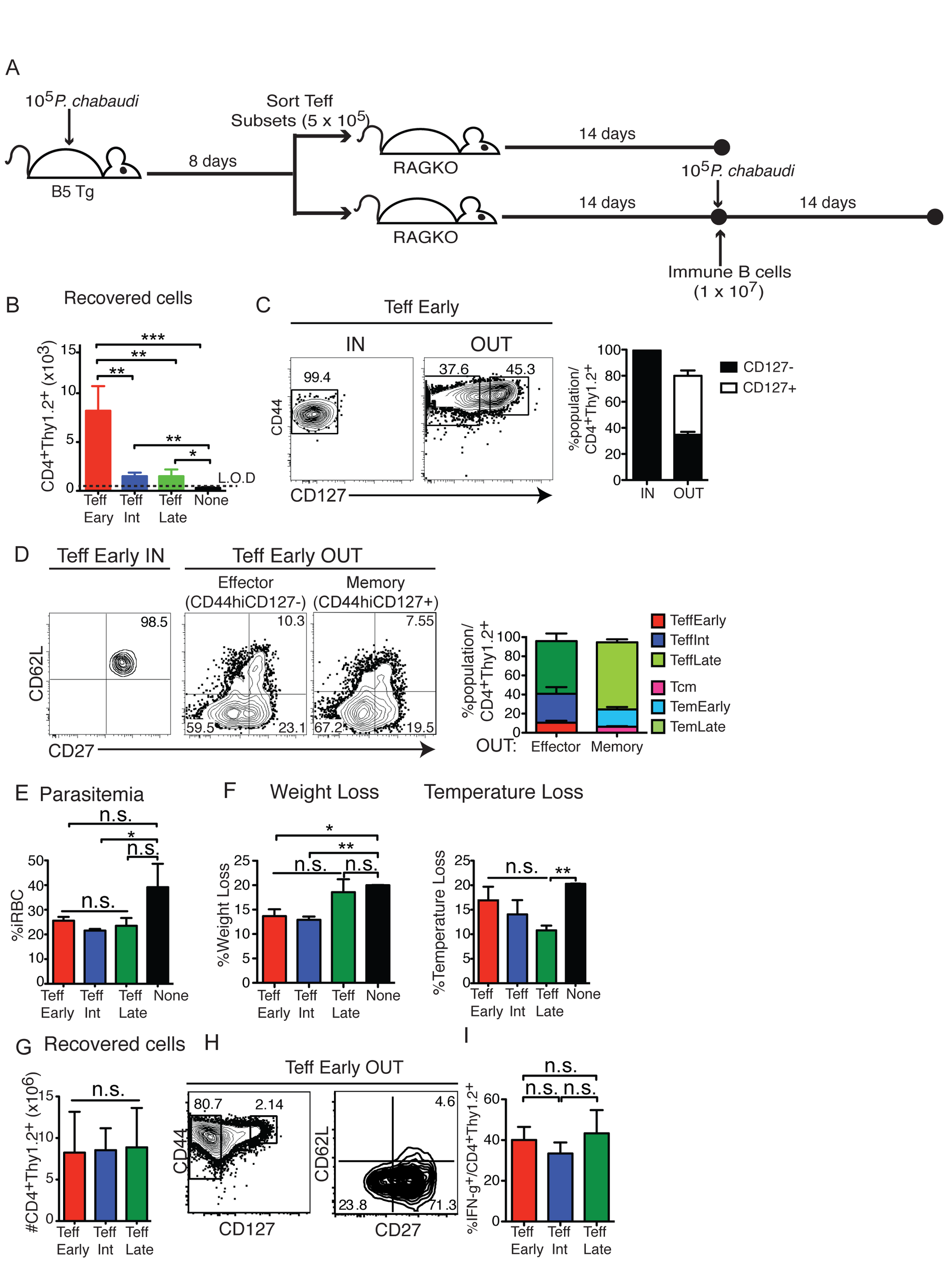

Supplement: S2 Fig — A) Schematic of experimental design. (B-I) Effector T cell subsets were sorted from the spleens of B5 TCR Tg at d8 p.i. and transferred (5x10^5^) into RAG2o mice for 14 days. (E-I) Fourteen days post-transfer, the RAG2o recipients were infected with P. chabaudi. B) Graph showing numbers of recovered B5 T cells (CD4+ Thy1.2+) per spleen in each group of recipients at day 14 post-transfer. (C, D) Contour plots and summary graphs showing transferred (IN) and recovered (OUT) TeffEarly (CD127- CD62Lhi CD27+) before infection. C) Proportions of Teff (CD127-) or Tmem (CD127hi) populations and D) Teff and Tmem subset phenotype (CD62L, CD27) of recovered B5 T cells on day 14 post-transfer are shown. (E-I) Infected recipient mice are shown at the peak of each symptom for each recipient (d8-10 p.i.). Graphs of E) Parasitemia (%iRBC/RBC), and F) percent weight loss and hypothermia are shown. G) Graph of average number of recovered B5 Tg T cells. H) Contour plot showing phenotype of Teff and subsets from TeffEarly OUT B5 T cells recovered from infected RAG mice, and I) Average Percent IFN-γ+ of B5 TCR Tg T cells recovered day 14 p.i. are shown. Contour plots are representative of 3 mice per group from 3 independent experiments. Data was analyzed in Prism using One-way ANOVA followed by Tukey’s and Students t-test for cell numbers. Error bars represent SEM, * p<0.05, ** p<0.01, *** p<0.001, and n.s.–not significant. (TIF) [file ppat.1006960.s002.tif]
